# Multiple intron motifs have redundant functions in the *trans*-splicing of the *mod(mdg4)* locus

**DOI:** 10.1093/nar/gkag823

**Published:** 2026-08-20

**Authors:** Iuliia Soldatova, Oguljan Beginyazova, Oksana Maksimenko, Yu-Jie Fan, Yong-Zhen Xu, Victoria Shender, Georgij Arapidi, Pavel Georgiev, Maxim Tikhonov

**Affiliations:** Department of the Control of Genetic Processes, Institute of Gene Biology Russian Academy of Sciences, 34/5 Vavilov St., 119334 Moscow, Russia; Department of the Control of Genetic Processes, Institute of Gene Biology Russian Academy of Sciences, 34/5 Vavilov St., 119334 Moscow, Russia; Center for Genome Research, Institute of Gene Biology Russian Academy of Sciences, 34/5 Vavilov St.,119334 Moscow, Russia; RNA Institute, State Key Laboratory of Virology, Hubei Key Laboratory of Cell Homeostasis, College of Life Sciences, TaiKang Center for Life and Medical Sciences, Wuhan University, Hubei 430072, China; RNA Institute, State Key Laboratory of Virology, Hubei Key Laboratory of Cell Homeostasis, College of Life Sciences, TaiKang Center for Life and Medical Sciences, Wuhan University, Hubei 430072, China; Lopukhin Federal Research and Clinical Center of Physical-Chemical Medicine of the Federal Medical and Biological Agency, Moscow 119435, Russia; Lopukhin Federal Research and Clinical Center of Physical-Chemical Medicine of the Federal Medical and Biological Agency, Moscow 119435, Russia; Department of the Control of Genetic Processes, Institute of Gene Biology Russian Academy of Sciences, 34/5 Vavilov St., 119334 Moscow, Russia; Department of the Control of Genetic Processes, Institute of Gene Biology Russian Academy of Sciences, 34/5 Vavilov St., 119334 Moscow, Russia

## Abstract

In contrast to canonical *cis-*splicing, *trans*-splicing combines exons from two distinct transcripts, yielding chimeric mRNAs. One striking example is the ubiquitously expressed *modifier of the mdg4* (*mod(mdg4)*) locus in *Drosophila*, where all mRNAs spanning over 30 isoforms are generated exclusively by *trans*-splicing. This process combines common constitutive exons with one of many alternative 3′ variable exons transcribed from independent promoters. Here, we demonstrate that *trans*-splicing is determined by a 600-bp region located in the proximal part of the last common intron. Unlike other known examples of *trans*-splicing, complementary sequences between donor and acceptor pre-mRNAs do not trigger *trans*-splicing of the *mod(mdg4)* gene. Deletion analysis revealed that the 600-bp intronic region contains multiple redundant motifs for RNA-binding proteins (RBPs) that collectively determine the level of *trans*-splicing. We identified specific binding of a large group of spliceosome-associated RBPs by affinity purification of the *in vitro* transcribed proximal part of the intronic RNA. A multifold reduction in mRNA expression caused by the partial removal of intronic sequences is partially compensated for by an increase in the translation efficiency of Mod(mdg4) isoforms. The results were consistent with a model in which multiple RBP motifs stabilize pre-mRNA binding to the spliceosome, thereby inducing *trans*-splicing.

## Graphical Abstract

**Graphical Abstract:**
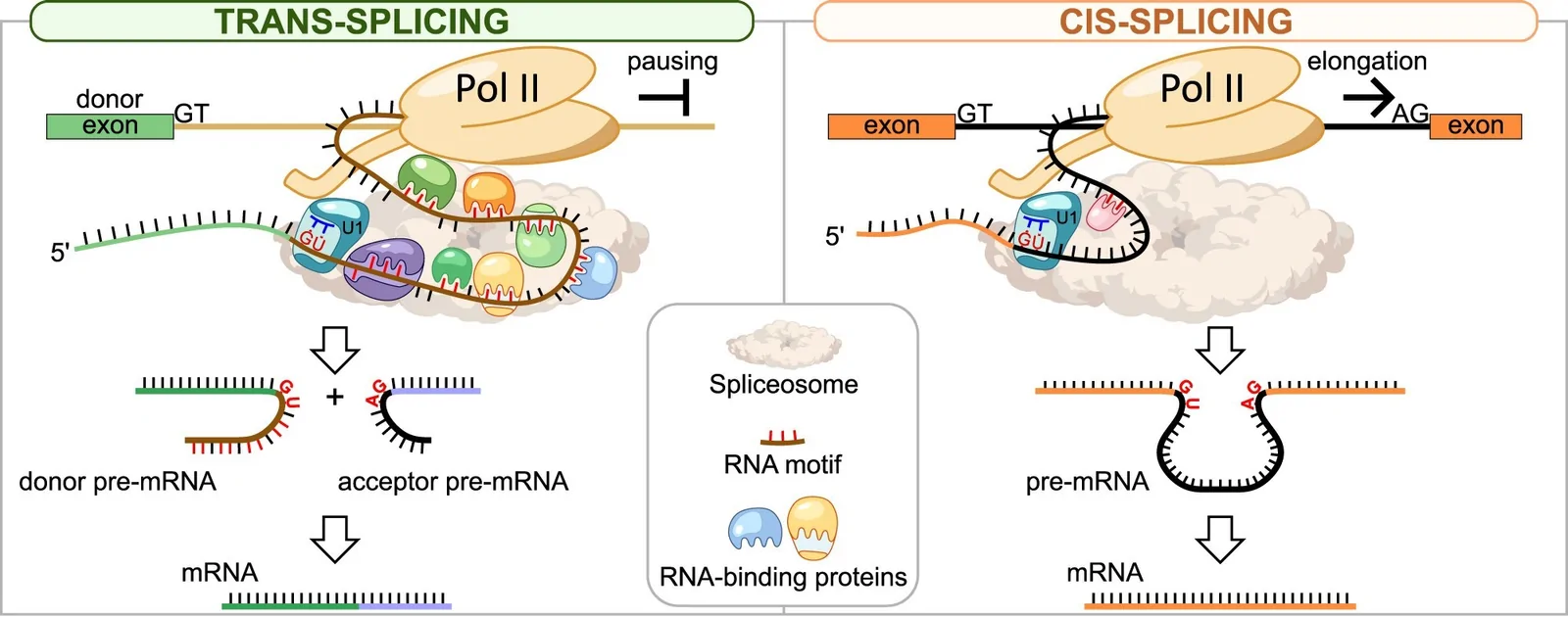

## Introduction

Most eukaryotic precursor messenger RNAs (pre-mRNAs) undergo splicing, a two-step process that requires the precise definition of intron–exon boundaries and comprises intron removal followed by exon ligation [1]. Splicing is a highly regulated process catalyzed by the spliceosome, a dynamic ribonucleoprotein complex comprising five noncoding U-rich small nuclear RNAs (snRNAs) and over 200 associated proteins [1–3]. The spliceosome plays a central role in identifying core regulatory sequences within pre-mRNAs and orchestrates a complex cascade of RNA–protein interactions. The U1 snRNP complex mediates initial recognition of the 5′ splice site of an intron through base-pairing interactions involving the 5′ end of U1 snRNA [4, 5]. This specific recognition mechanism does not rely solely on the primary sequences of the splice sites, as their consensus features are rather rudimentary. Instead, specific motifs within exons and introns adjacent to donor and acceptor sites interact with RNA-binding proteins (RBPs), thereby ensuring efficient spliceosome assembly and splicing [3].

More than 200 RBPs have been annotated in *Drosophila* [6, 7]. The predominant class of *Drosophila* RBPs includes one or several copies of a conserved RNA-binding domain (RBD) [8, 9] that typically recognizes short and degenerate motifs. In addition to RBDs, these proteins often contain auxiliary domains, such as serine/arginine-rich (SR) domains, K-homology (KH) domains, and zinc fingers [10–13]. While some RBPs are ubiquitously expressed and contribute to constitutive splicing, others exhibit highly tissue-specific expression patterns. These specialized RBPs play a key role in triggering alternative splicing and, hence, the synthesis of distinct protein isoforms [10, 12, 13]. RBPs bind to *cis-*regulatory RNA elements, termed splicing enhancers or silencers, that modulate the efficiency of donor- and acceptor-site utilization during alternative splicing [1, 14–16].

Alternative splicing is widely regarded as a major driver of proteome diversity, generating multiple mRNA variants from a single pre-mRNA through the differential selection of splice sites [14, 17]. Here, we focus on a highly specialized form of alternative splicing known as *trans*-splicing, in which exons from distinct pre-mRNAs that originate either from the same genomic locus (intragenic *trans*-splicing) or from completely different genes (intergenic *trans*-splicing) are joined [18–20]. Despite its remarkable capacity to generate transcript diversity, *trans*-splicing is normally repressed to prevent the formation of aberrant or deleterious chimeric mRNAs. Nevertheless, functional *trans*-splicing has been documented in numerous higher eukaryotes, including humans [21–23]. Current models suggest that long complementary sequences typically mediate the pairing of distinct transcripts to facilitate *trans*-splicing [20, 21, 24]. For example, complementary base pairing brings pre-mRNAs from different *sDscamβ* clusters of *Tetranychus urticae* into proximity, enabling the assembly of chimeric transcripts and expanding isoform diversity [25].

**Figure 1.**
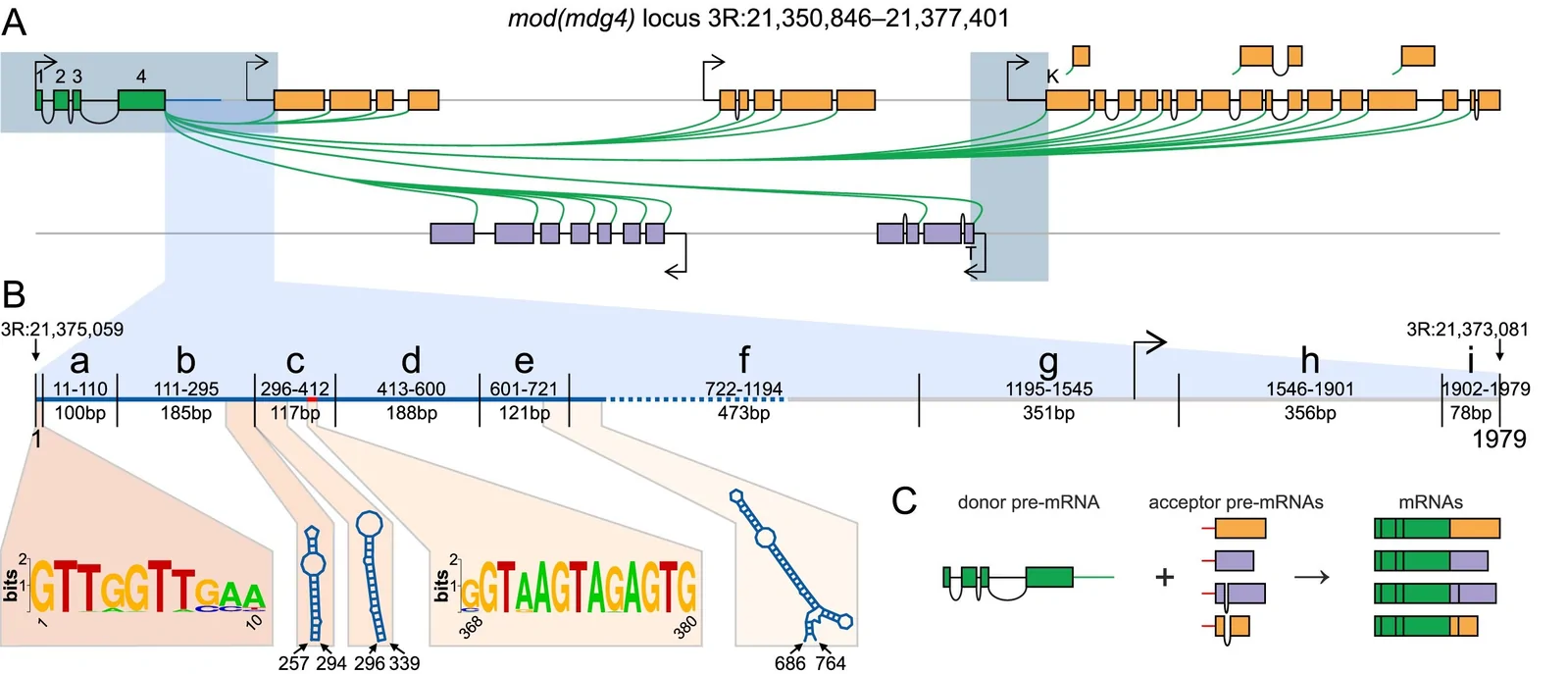
Organization of the *mod(mdg4)* locus and an illustration of *trans*-splicing. (**A**) The structure of the *mod(mdg4)* locus in *D. melanogaster*. Arrows indicate annotated promoters. Constitutive exons shared by all isoforms are shown in green and are numbered from 1 to 4. Alternative exons located on the same DNA strand as the constitutive exons are shown in orange, and those located on the opposite strand are shown in purple. Black arcs indicate constitutive introns. *Trans*-splicing events connecting constitutive and alternative exons are shown by green curves. The gray boxes indicate genomic fragments used to generate the donor and acceptor transgenes employed in this study. The alternative exons corresponding to the T and K isoforms used in the acceptor construct are indicated. The scheme is based on the FlyBase annotation of the *mod(mdg4)* locus [36–38], and genomic coordinates are according to the *D. melanogaster* reference genome assembly dm6 [39]. Schematic representation adapted from Beginyazova et al. [40] under the CC BY 4.0 license. (**B**) Schematic representation of the fourth intron of *mod(mdg4)*. The intron is subdivided into regions (a–i); genomic coordinates (dm6), coordinates relative to the beginning of the intron, and fragment lengths are indicated above the proportional intron map. Functionally important sequence elements are shown below, including the conserved 5′ region [41], the predicted RNA secondary structures located at the end of region *b* and the beginning of region *c* [42], the conserved *c^13^* motif required for efficient *trans*-splicing [34], and the RNA secondary structure associated with noncanonical transcription termination [32]. (**C**) Schematic representation of mature mRNA formation by *trans*-splicing. The constitutive region of the locus produces the donor pre-mRNA (green), whereas the alternative regions generate multiple acceptor pre-mRNAs (orange and purple). *Trans*-splicing between donor and acceptor pre-mRNAs produces 31 mature mRNA isoforms, each containing four constitutive exons fused to one or two alternative exons.

The *mod(mdg4)* locus of *Drosophila* represents a unique paradigm in which *trans*-splicing generates 31 diverse mRNAs encoding isoforms with distinct C-terminal domains [26, 27]. The *mod(mdg4)* locus (Fig. 1A) contains a common region encoding the constant core of the Mod(mdg4) proteins. This region harbors an N-terminal BTB domain and alternative 3′ exons organized into five clusters, each transcribed from an independent promoter. The well-characterized Mod(mdg4)-67.2 isoform contains a unique C-terminal domain that specifically interacts with the architectural protein Su(Hw). This complex is involved in organizing chromosomal architecture, insulation, and the direct silencing of promoters [28–31]. The *mod(mdg4)* locus is exceptional because all of its mature mRNAs are produced exclusively via *trans*-splicing [32, 33]. To date, no discernible complementary sequences have been identified between the introns involved in *trans*-splicing at this locus [33–35]. Consequently, the assembly of alternative *mod(mdg4)* mRNAs must proceed via a novel mechanism that couples the 5′ and 3′ splice sites of transcripts derived from independent promoters.

Several *cis-*regulatory elements critical for *trans*-splicing have been mapped to the proximal region of the fourth (and last) common intron of the *mod(mdg4)* gene (Fig. 1B). A conserved 13-bp motif capable of robustly recruiting the U1 snRNP through strong base pairing [34] and a conserved sequence flanking the donor site [41] contribute to *trans*-splicing. However, their *in vivo* deletion only marginally impairs *trans*-splicing efficiency [41, 33]. In contrast, deletion of a 73-bp sequence encompassing this 13-bp core severely reduces *trans*-splicing *in vivo*, suggesting that the process is driven by multiple distinct motifs acting in concert. There is a conserved sequence located distally within the intron that forms a stable secondary RNA structure [34] and is involved in the termination of transcription without mRNA polyadenylation [32, 33]. Notably, RNA polymerase II (RNA pol II) pauses within the proximal part of this fourth intron, with no transcription having been detected in its distal region [33].

In this study, we mapped the specific sequences and motifs that govern *trans*-splicing at the *mod(mdg4)* locus using a previously established model system [33] in combination with *in vivo* genome editing via CRISPR–Cas9 and the φC31 integrase system [43–47]. We demonstrate that a 600-bp region of the fourth intron contains multiple, redundant RBP motifs that cooperate to produce efficient *trans*-splicing. RNA affinity purification analysis revealed three sub-complexes, within which five distinct RBPs possess specific binding sites inside this critical *trans*-splicing-determining region. Furthermore, we show that partial disruption of *trans*-splicing, which leads to a multifold reduction in *mod(mdg4)* isoform transcripts, does not compromise mutant viability, even though *mod(mdg4)* is an essential gene in *Drosophila*. Finally, we demonstrate that a severe reduction in transcripts encoding the Mod(mdg4)-67.2 isoform—a critical component of the Su(Hw) insulator complex—does not notably diminish total Mod(mdg4) protein levels.

## Materials and methods

### Construct design and molecular cloning

Genetic constructs were prepared following standard molecular biology protocols, utilizing reagents from Thermo Fisher Scientific (Waltham, MA, USA). Plasmid propagation occurred in *Escherichia coli* DH5α under appropriate selective conditions. Site-specific integration constructs were developed from a pBluescript-derived backbone, incorporating *mini-white*, a loxP site, a multiple cloning site (MCS), and an attB site for φC31 integrase-mediated insertion. Promoters were obtained from their respective sources: the *mod(mdg4)* promoter was amplified by polymerase chain reaction (PCR) from *Drosophila melanogaster* genomic DNA, the *actin5C* promoter was excised from pAc5.1/V5-His, and the UAS promoter was obtained from pUASTm [48]. Genomic DNA was used as the template for PCR amplification of coding sequences for *mod(mdg4*) and *grip*, and the *reaper* (*rpr*) internal ribosome entry site (IRES). Intact introns were amplified directly, and deletions or site-specific mutations were subsequently introduced via fusion PCR or by fragment excision between restriction sites. Promoters, outrons, and short 5′ exon fragments for the acceptor constructs were also amplified from genomic DNA. Firefly luciferase (*Fluc*) was excised from pGL3-Basic (Promega), while mCherry was PCR-amplified from a laboratory stock vector. The SV40 polyadenylation signal was obtained from pAc5.1/V5-His and inserted downstream of the marker genes. sgRNAs for targeted mutagenesis of *mod(mdg4)* intron 4, targeting the *b* and *d* regions, were selected using the Fly CRISPR Optimal Target Finder [44]. Two sgRNAs were cloned into a dual U6 promoter vector based on pCFD4-U6:1_U6:3 [43] (Addgene #49411) for simultaneous expression. The homology-directed repair donor template comprised PCR-amplified genomic fragments, the mCherry coding sequence, the 3xP3 promoter (both from laboratory plasmids), and the SV40 polyadenylation signal, which were from pAc5.1/V5-His. attP recombination sites were incorporated at the primer 5′ ends. Sequences for targeted integration into the fourth intron landing platform were PCR-amplified with attB-containing primers and then cloned into a laboratory vector carrying the 3xP3-driven eGFP reporter gene.

The oligonucleotides used are listed in Supplementary Table S2

### *Drosophila* stocks, transgenesis, and genetic crosses

*D. melanogaster* stocks were maintained at 25°C under standard conditions. Transgenic constructs were integrated site-specifically into the 22A2 locus using φC31 integrase-mediated recombination. Plasmid DNA (200 ng/μl) was microinjected into preblastoderm embryos [49] from a fly line (Bloomington Stock Center #24 481: *y^1^ M{vas-int.Dm}ZH-2A w^1118^; M{3xP3-RFP.attP’}ZH-22A*) [50] that carries an attP docking site and expresses φC31 integrase under the germline-specific *vasa* promoter. F0 flies were crossed with *y^1^w^1118^*, and F1 progeny with the transgene were selected via *mini-white* eye pigmentation. Homozygous lines were established by crossing with *y^1^w^1118^; Kr^If−1^*/SM5. Integration was confirmed via PCR across transgene–genome junctions and sequencing of selected products.

To assess the effects of different *mod(mdg4)* mutant alleles on the *ct^6^* allele phenotype, we generated specific genotypes using a three-step crossing scheme. First, *y^1^ w^1118^ ct^6^*; MKRS/TM6B females were crossed with y^1^ w^1118^; mod(mdg4)*/mod(mdg4)* males, where mod(mdg4)* represents various mutant alleles. The resulting *y^1^ w^1118^ ct^6^*/Y; mod(mdg4)*/TM6B males were then crossed with *y^1^ w^1118^ ct^6^*; MKRS/TM6B females. For the final cross, y^1^ w^1118^ ct^6^; mod(mdg4)*/TM6B females from this generation were crossed with *y^1^ w^1118^ ct^6^*/Y; mod(mdg4)*/TM6B males. Wings from the desired *y^1^ w^1118^ ct^6^*/Y; mod(mdg4)*/mod(mdg4)* males were subsequently dissected and imaged using a Nikon SMZ18 stereomicroscope with a Nikon DS-Ri2 camera (Tokyo, Japan).

### Genome editing within the *mod(mdg4)* locus *in vivo*

Microinjection of the plasmid mixture (500 ng/μl) was performed on *D. melanogaster* embryos that express Cas9 under the ubiquitous *actin5C* promoter (BDSC #58492). The resulting F0 flies were crossed with *y^1^w^1118^* flies. F1 progeny demonstrating successful gene editing, as evidenced by *mCherry* expression, were then crossed with *y^1^w^1118^*; MKRS/TM6B to establish the *y^1^w^1118^; mod(mdg4)^attPx2^*/TM6B line. Subsequent successive crosses facilitated the creation of *y^1^ w^1118^* P(y[+t7.7]=nanos-phiC31\int.NLS)X; *mod(mdg4)^attPx2^*, which expresses φC31 integrase driven by the maternal *nanos* promoter.

Plasmid DNA containing *mod(mdg4)* intron fragments, flanked by attB sites (200 ng/μl), was microinjected into embryos of the established platform line. The resulting F0 flies were crossed with *y^1^w^1118^*; MKRS/TM6B. Transgenic progeny were screened based on the loss of *mCherry* fluorescence (indicating successful replacement of the target locus) and the absence of *eGFP* (confirming specific integration of only the desired fragment, not the plasmid backbone). Carriers of the integration were then crossed to MKRS/TM6B to remove the X-linked φC31 integrase. Integration was subsequently confirmed by PCR amplification and sequencing of the genomic region adjacent to the insertion site.

### Quantification of splicing efficiency and gene expression

Biological replicates, consisting of 15–30 adult males (2- to 3-day-old), were flash-frozen in liquid nitrogen. Adult males were used because expression measurements in adult females are strongly influenced by the high background signal from the ovaries and developing oocytes, making it difficult to distinguish expression in other tissues. This choice also ensured consistency with our previous analyses of *mod(mdg4)* expression [33]. Samples were homogenized in 200 µl of TRI Reagent^®^ (MRC, Cincinnati, OH, USA), brought to a final volume of 1 ml, and total RNA was extracted according to the manufacturer’s protocol. RNA was then treated with DNase I (Thermo Fisher Scientific, Waltham, MA, USA) to eliminate residual genomic DNA. Complementary DNA (cDNA) synthesisc utilized random hexamer primers and was carried out using RevertAid, Maxima Reverse Transcriptase (both Thermo Fisher Scientific), or LunaScript^®^ RT Master Mix (Primer-free; New England Biolabs, Ipswich, MA, USA), following the respective manufacturer’s guidelines. For quantitative PCR, Diamant TaqA polymerase (BelBioLab, Moscow, Russia) was used with either EvaGreen dye (Biotium, Inc., Fremont, CA, USA) or TaqMan probes (Lumiprobe, Moscow, Russia) (using ROX as a reference dye) on a QuantStudio^™^ 6 Flex Real-Time PCR System (Thermo Fisher Scientific). Relative mRNA levels were quantified through standard curves derived from serial dilutions of genomic DNA (for housekeeping genes) or PCR products spanning splice junctions (for splicing efficiency). Student’s *t*-test was used to assess statistical significance. The MIQE guidelines [51] are provided in Supplementary Table S3.

### Fly extract preparation

Twenty adult flies were cooled in liquid nitrogen. Homogenization was performed for 30 s with a pestle in 200 μl of extraction buffer (20 mM 4-(2-hydroxyethyl)-1-piperazineethanesulfonic acid (HEPES), pH 7.5, 100 mM KCl, 5% glycerol, 10 mM ethylenediaminetetraacetic acid (EDTA), 1% NP-40, 1% sodium deoxycholate, 0.1% sodium dodecyl sulfate (SDS), 1 mM dithiothreitol (DTT), 5 mM phenylmethylsulfonyl fluoride (PMSF), and 1:100 Calbiochem Complete Protease Inhibitor Cocktails VII and V). The resulting suspension was incubated on ice for 10 min. Subsequently, the sample underwent sonication in a Bioruptor (Diagenode, USA) for 3 min at setting H, utilizing 15-s ON and 45-s OFF cycles. Following this, 4× SDS–PAGE sample buffer was added to the homogenate. The extracts were heated to 100°C for 10 min, centrifuged at 16 000 × *g* for 5 min, and then loaded onto a 10% SDS–PAGE gel. Immunoblot analysis employed rabbit antibodies against the C-terminal domain of Mod(mdg4)-67.2 and mouse monoclonal anti-lamin Dm0 (clone ADL84.12, #ADL84.12, DSHB, USA).

### Target enrichment and RNA sequencing

Total RNA was extracted from imago males of the control *y^1^w^1118^* line and the *y^1^w^1118^; mod(mdg4) ^c13^/mod(mdg4)^c13^*line. An aliquot of 50 µl of hydrophilic streptavidin magnetic beads (NEB) were washed with 500 µl of wash/binding buffer (0.5 M NaCl, 20 mM Tris–HCl pH 7.5, 1 mM EDTA), magnetically pelleted, and subsequently blocked with 100 µl of wash/binding buffer containing 250 ng/µl of sonicated salmon sperm DNA. After an additional wash with 500 µl of wash/binding buffer, the beads were incubated for 15 min at room temperature, with rotation, in 25 µl of wash/binding buffer containing 200 pmol of a biotinylated oligonucleotide complementary to exon 4 (8 pmol/µl) RNA. This incubation was followed by two subsequent washes with 100 µl of wash/binding buffer.

Total RNA (100 µg) was denatured at 65°C, chilled on ice, and then added to the beads. This mixture was incubated at 55°C for 30 min with agitation. Five sequential washes followed. The first two washes used wash buffer (1 M NaCl, 20 mM Tris–HCl pH 7.5, 1 mM EDTA, and 0.05% Tween-20) at 55°C. The third wash employed the same buffer but without Tween-20. The fourth wash used low salt buffer [150 mM NaCl, 20 mM Tris–HCl (pH 7.5), and 1 mM EDTA], and the fifth was with ice-cold low salt buffer. RNA was eluted by adding 200 µl of TRI Reagent^®^ and then extracted following the manufacturer’s protocol with glycogen added prior to isopropanol precipitation.

Preparation of RNA-seq libraries followed the manufacturer’s instructions for the NEBNext^®^ Ultra RNA Library Prep kit (NEB, USA). Amplified libraries underwent quantification by fluorometry using a DS-11 (DeNovix, USA) and a Bioanalyzer 2100 (Agilent, USA). Diluted libraries were subsequently clustered on a pair-read flowcell and sequenced on an Illumina NovaSeq 6000 system (USA). Alignment of sequencing reads to the *D. melanogaster* dm6 genome [39] was performed using STAR v2.7 [52], which simultaneously assessed splice junction coverage.

### Prediction of intermolecular RNA–RNA interactions

To identify potential intermolecular *trans*-interactions within the transcribed RNA, we employed a sliding-window approach combined with thermodynamic folding algorithms. The full-length transcripts of the *mod(mdg4)* locus was systematically partitioned into overlapping 300-nucleotide windows with a step size of 50 nt.

Pairwise RNA–RNA interactions between all generated windows were predicted using RNAcofold from the ViennaRNA Package [53]. This algorithm models the co-folding of two distinct RNA molecules and calculates the hybridization free energy while explicitly accounting for both intramolecular secondary structures and the accessibility of potential binding sites. The folding temperature was set to 25°C, and base-pairing probability estimates were enabled.

The resulting predictions were subjected to a stringent multistep filtering procedure. Interactions were retained for downstream analysis only if they satisfied all of the following criteria: (i) a predicted binding free energy Δ*G* ≤ −10 kcal/mol, (ii) an individual base-pairing probability > 0.7, (iii) >20 intermolecular base pairs, and (iv) detection in more than half of all relevant sliding-window predictions, defined as the interaction being predicted in >50% of the window pairs in which the corresponding interaction could be identified.

The filtered intermolecular interaction networks were visualized using Circos software [54].

### Cell fractionation and nuclear extract preparation

*D. melanogaster* S2 cells were seeded in SFX medium (HyClone, Logan, UT, USA) and cultured at 25°C. For each pulldown, 10^7^ cells were collected, washed with ice-cold PBS, and homogenized with a Dounce homogenizer (tight pestle, 20 strokes) in lysis buffer (20 mM Tris–HCl 7.5, 10 mM KCl, 10 mM MgCl_2_, 2 mM EDTA, 10% glycerol, 1% Triton X-100, 1 mM DTT, 1 mM PMSF, and 1× Halt Protease Inhibitor Cocktail). The homogenate was incubated on ice for 10 min and then centrifuged at 3000 × *g* to separate the cytoplasmic fraction from the nuclei. The nuclear pellet was resuspended in the same lysis buffer and sonicated in a Bioruptor (Diagenode, USA) for 3 min using the “L” setting (30-s ON/30-s OFF). NaCl was added to a final concentration of 500 mM, and the suspension was incubated on ice for an additional 60 min with periodic mixing. To reduce viscosity, sonication was repeated as above. Cell debris was pelleted by centrifugation at 10 000 × *g* for 30 min at 4°C. The supernatant (nuclear extract) was then diluted to 250 mM NaCl with incubation buffer-0 (20 mM Tris–HCl, pH 7.5, 10 mM MgCl_2_, 10% glycerol, 0.1% NP-40, 0.5 mM PMSF, 2 mM Ribonucleoside Vanadyl Complex, and 1× Halt Protease Inhibitor Cocktail) and kept for subsequent incubation with RNA–Streptavidin magnetic beads.

### RNA biotinylation

Two different RNAs (*abc* and *cba*) were transcribed *in vitro* from a T7 promoter using the Megascript kit (Ambion), according to the manufacturer’s instructions. For biotinylation, 1 μg of each RNA was mixed with 50 mM Tris–HCl pH 7.8, 10 mM MgCl₂, 1 mM DTT, 1 mM ATP, 10% DMSO, 15% PEG, 1 μM pCp biotin, 10 U T4 RNA ligase, and 50 U RNase inhibitor. The reaction was incubated overnight at 16°C. Biotinylated RNA was purified by chloroform:isoamyl alcohol (24:1) extraction and verified by dot blot analysis.

### Immobilization of biotinylated RNA on streptavidin beads

Dynabeads MyOne Streptavidin T1 magnetic beads (Thermo Fisher Scientific) (20 µl per sample) were washed twice with buffer A (100 mM NaOH and 50 mM NaCl), once with buffer B (100 mM NaCl), and twice with buffer C (20 mM Tris–HCl pH 7.5). The beads were then resuspended in RNA-binding buffer (20 mM Tris–HCl pH 7.5, 1 M NaCl, and 1 mM EDTA). Biotinylated RNA was heated at 90°C for 2 min and immediately placed on ice, then added to the bead suspension and incubated at room temperature for 30 min. RNA-coated beads were washed twice with buffer C and once with RNA–protein buffer (20 mM Tris–HCl pH 7.5, 50 mM NaCl, 2 mM MgCl2, 0.1% Tween-20).

### RNA pull down

For the binding reaction, 300 μl of nuclear extract (diluted to 250 mM NaCl) was mixed with 150 μl of 50% glycerol and 50 μl of 10× RNA–protein buffer (200 mM Tris–HCl pH 7.5, 500 mM NaCl, 2 mM MgCl₂, and 1% Tween-20). This mixture was added to the RNA-coated beads and incubated overnight at 4°C with gentle rotation. After three washes with wash buffer (10 mM Tris–HCl pH 8.0, 150 mM NaCl, and 2 M urea), bound proteins were eluted with elution buffer (10 mM Tris–HCl pH 8.0, and 8 M urea).

### In-gel solution protein digestion

Protein concentrations in biological samples after RNA affinity purification were determined Quick Start Bradford protein assay (Bio-Rad), according to the manufacturer’s standard protocol (bovine serum albumin was used as the standard). Disulfide bonds were reduced with DTT in 5 mM final concentration for 30 min at room temperature (RT). Afterward, iodoacetamide was added to a final concentration of 10 mM. The samples were incubated at RT for 20 min in the dark, and the reaction was stopped by adding DTT to a final concentration of 5 mM for 20 min. Then, samples was diluted with ammonium bicarbonate solution to reduce urea concentration to 4 M. LysC (Promega) was added at the ratio of 1:100 w/w and the samples were incubated for 4 h at 37°C. Then, samples were diluted with ammonium bicarbonate solution to reduce urea concentration to 2 M, trypsin (Promega) was added at the ratio of 1:100 w/w, and the samples were incubated for 14 h at 37°C. After 14 h, the reaction was stopped by the addition of trifluoroacetic acid (TFA) to the final concentration of 0.5%. The tryptic peptides were desalted using SDB–RPS membrane (Sigma–Aldrich) and vacuum-dried. Prior to LC–MS/MS analysis, samples were redissolved in 5% acetonitrile (ACN) with 0.1% TFA solution and sonicated.

### LC–MS/MS analysis

Proteomic analysis was performed using a Q Exactive HF mass spectrometer. Samples were loaded onto 50-cm columns packed in-house with C18 3 μM Acclaim PepMap 100 (Thermo Fisher), with an Ultimate 3000 Nano LC System (Thermo Fisher) coupled to the MS (Q Exactive HF, Thermo Fisher). Peptides were loaded onto the column thermostatically controlled at 40°C in buffer A (0.2% formic acid) and eluted with a linear (120 min) gradient of 4%–55% buffer B (0.1% formic acid and 80% acetonitrile) in A at a flow rate of 350 nl/min. Mass spectrometric data were stored during automatic switching between MS1 scans and up to 15 MS/MS scans (topN method). The target value for MS1 scanning was set to 3 × 10^6^ in the range 300–1200 *m/z* with a maximum ion injection time of 60 ms and a resolution of 60 000. The precursor ions were isolated at a window width of 1.4 *m/z* and a fixed first mass of 1000 *m/z*. Precursor ions were fragmented by high-energy dissociation in a C-trap with a normalized collision energy of 28 eV. MS/MS scans were saved with a resolution of 15 000 at 400 *m/z* and at a value of 1 × 10^5^ for target ions in the range of 200–2000 *m/z* with a maximum ion injection time of 30 ms.

### Protein identification and spectral counting

Raw data files acquired on the Q Exactive HF-X were converted to Mascot generic format (mgf) peak lists using MSConvert (ProteoWizard Software Foundation) with the following parameters: “–mgf –filter peakPicking true [1,2].” The resulting peak lists were searched against the UniProt Knowledgebase (taxon 7227 *D. melanogaster*; downloaded 13/04/2026 with 44 174 protein sequences) concatenated with a reversed decoy set using MASCOT (version 2.5.1, Matrix Science Ltd., UK) and X! Tandem (ALANINE, 2017.02.01, The Global Proteome Machine Organization). For both search engines, precursor and fragment mass tolerances were set to 20 and 50 ppm, respectively. Database-searching parameters included the following: tryptic digestion with one possible missed cleavage, static modification for carbamidomethyl (C), and dynamic/flexible modifications for oxidation (M). For X! Tandem we also selected parameters that allowed a quick check for protein N-terminal residue acetylation, peptide N-terminal glutamine ammonia loss, or peptide N-terminal glutamic acid water loss. Result files were submitted to Scaffold 5 software (version 5.1.0) for validation and meta-analysis. We used the local false discovery rate scoring algorithm with standard experiment-wide protein grouping. For the evaluation of peptide and protein hits, a false discovery rate of 5% was selected for both. False positive identifications were based on reverse database analysis. The mass spectrometry proteomics data have been deposited to the ProteomeXchange Consortium via the PRIDE [55] partner repository with the dataset identifier PXD079014 and 10.6019/PXD079014.

## Results

### The role of the proximal region of the fourth intron in *trans*-splicing

**Figure 2.**
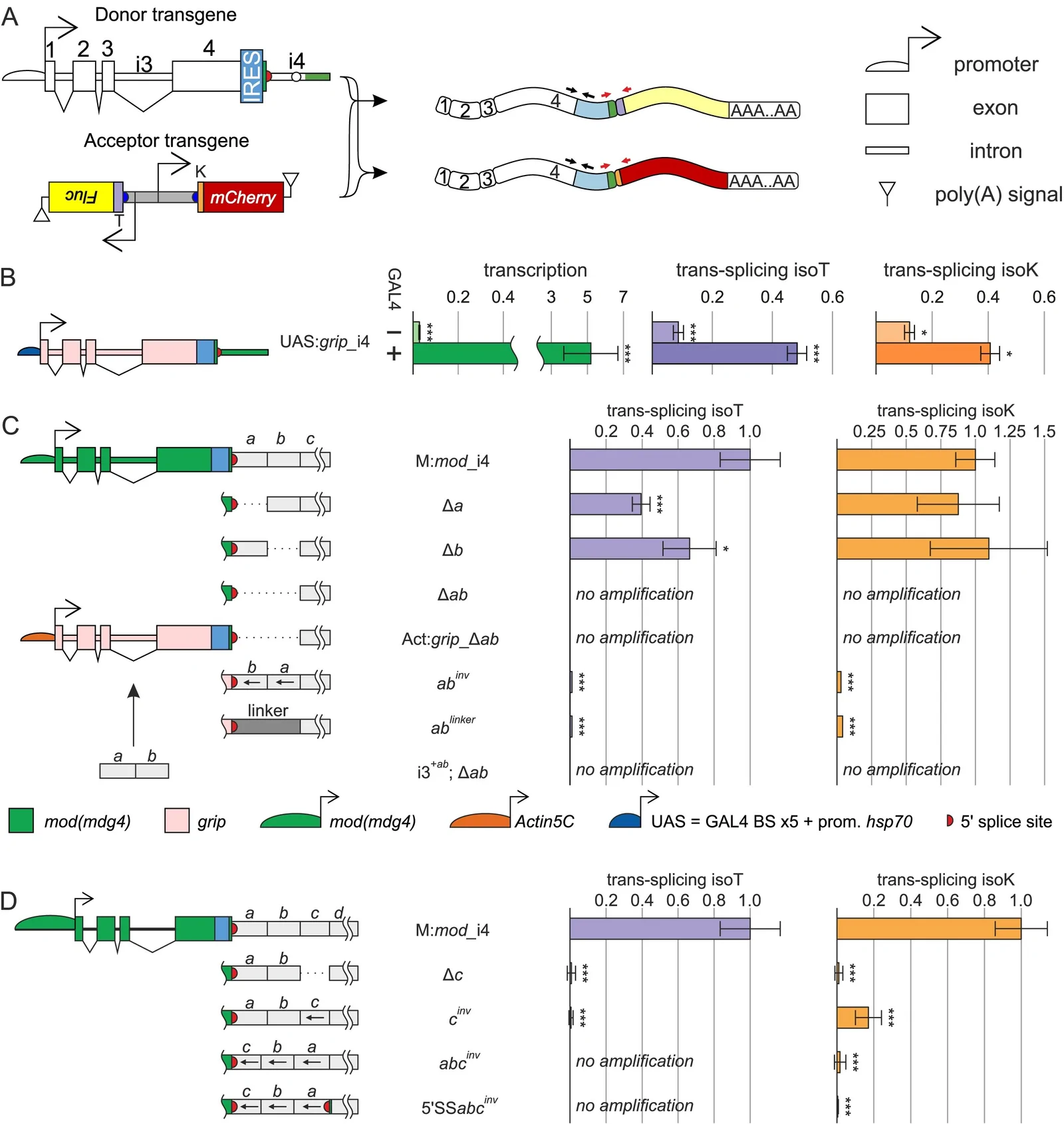
Functional analysis of the proximal region of intron 4 in *trans*-splicing of *mod(mdg4)*. (**A**) Schematic representation of the model *trans*-splicing system. The donor transgene (top) contains the *mod(mdg4)* promoter (arrows and semicircles), constitutive exons (rectangles), introns (lines), an IRES sequence, and intron 4 with the 5′ splice donor site (the red semicircle). The acceptor transgene (bottom) contains the bidirectional promoter associated with the T and K isoforms, together with their corresponding outrons (gray lines). Coding regions were replaced with the *Fluc* (yellow) and *mCherry* (red) reporter genes. Transcription produces one donor pre-mRNA and two alternative acceptor pre-mRNAs. *Trans*-splicing generates two mature mRNA isoforms consisting of the common 5′ region fused to either the T- or K-specific 3′ region. Red and black arrows indicate primer positions used to quantify *trans*-splicing products and donor transcript levels, respectively. (**B**) *Trans*-splicing supported by a donor construct containing only intron 4 and a short fragment of exon 4 from *mod(mdg4)*. The chimeric donor transgene consists of the UAS promoter and a *grip*-derived gene body retaining only intron 4 from *mod(mdg4)*. Relative donor transcription levels and *trans*-splicing efficiencies for the T and K isoforms are shown on the right. (**C**) The effects of deletions and inversions within regions *a* and *b* on *trans*-splicing efficiency. The schematics of the donor constructs are shown on the left. The donor segment is driven by either the endogenous *mod(mdg4)* promoter (green), the *Act5C* promoter (orange), or the UAS promoter (blue), and contains gene-body sequences derived from either *mod(mdg4)* (green) or *grip* (pink). The splice donor site is indicated by the red semicircle. Deletions are shown as dashed lines and inversions as black arrows. The dark gray segment corresponds to a control intronic fragment derived from *ATPsynC*. The construct labeled “i3^+ab^; Δab” contains the a–b region inserted into intron 3 of the *grip* gene derivative. Construct names are centrally located. The relative *trans*-splicing efficiencies for the T and K isoforms are shown on the right. (**D**) The effects of inversion of region *c* or the entire *abc* fragment on *trans*-splicing efficiency. Construct organization and graphical annotations are as described in panel (C). Each *trans*-heterozygous combination was analyzed in at least three biological replicates. Error bars represent standard deviations (*n* = 3–13). Statistical significance was assessed relative to the corresponding control: *P < 0.05, **P < 0.01, ***P < 0.001.

Previous studies have demonstrated that the fourth intron of the *mod(mdg4)* gene plays a key role in *trans*-splicing [34, 33, 40]. To determine whether the fourth intron alone is sufficient to induce *trans*-splicing, we used a previously established model system consisting of donor and acceptor transgenes integrated into the 22A genomic locus (Fig. 2A and Supplementary Fig. S1). The reference donor construct contained the *mod(mdg4)* promoter and the gene body comprising four exons and three introns (M:mod_i4). An IRES sequence from the *reaper* gene was inserted downstream of exon 4 to enable specific RT-PCR detection. In the M:mod_i4 donor, the IRES was followed by a 40 bp fragment of exon 4 and the fourth intron (*i4*) of *mod(mdg4)*. The acceptor transgene contained the bidirectional promoter that, in the endogenous locus, drives transcription of the T and K isoforms, together with their corresponding outrons, while the coding regions were replaced with *Fluc* and *mCherry* reporters (F-mC) (Fig. 2A).

We generated a chimeric donor construct composed predominantly of heterologous sequences to determine whether the fourth intron alone was sufficient to induce *trans*-splicing. Specifically, we used a chimeric UAS promoter consisting of five binding sites for the yeast transcriptional activator GAL4 and the minimal *hsp70* promoter core that drives weak ubiquitous transcription in the absence of GAL4 activation. We inserted a fragment of the *grip* gene downstream of the promoter that featured an exon-intron organization comparable to that of *mod(mdg4)* [40] (UAS:grip_i4; Fig. 2B and Supplementary Fig. S2). In this construct, only the fourth intron of *mod(mdg4)* and a short fragment of exon 4 were retained.

The *trans*-splicing efficiency observed in heterozygous M:mod_i4/F-mC males was defined as the reference value of 1. In UAS:grip_i4/F-mC males, *trans*-splicing efficiency was reduced to approximately 10% of the control level (Fig. 2B), consistent with the substantially lower transcriptional activity of the UAS promoter relative to the endogenous *mod(mdg4)* promoter. To increase transcription from the UAS promoter, we introduced an *Act5C-GAL4* transgene (Bloomington Drosophila Stock Center #4414) into the UAS:grip_i4/F-mC background by genetic crossing (Fig. 2B). Activation of GAL4 increased transcription from the UAS promoter by approximately 30-fold, resulting in a corresponding 4–5-fold increase in *trans*-splicing efficiency. Because the UAS:grip_i4 donor retains only the fourth intron of *mod(mdg4)*, these results indicate that sequences within this intron are sufficient to promote *trans*-splicing.

The *cis-*elements required for *trans*-splicing are expected to reside within the proximal 600 bp of the fourth intron, as transcription is interrupted downstream at a conserved termination site (Fig. 1B) [32]. Previous studies have focused on a 300–450 bp interval corresponding to TSA/region *c* that contains the conserved 13 bp motif (*c^13^*). Although deletion of this region substantially reduced *trans*-splicing efficiency, it did not completely abolish it, suggesting the existence of additional regulatory elements within the intron. In a previous study, we subdivided the proximal 600 bp region of intron 4 into four segments: region *a* (11–108 bp), region *b* (109–295 bp), region *c* (296–412 bp), and region *d* (413–600 bp) (Fig. 1B) [33]. Deletion of either region *a* or *b* individually only slightly reduced *trans*-splicing efficiency. In this study, we repeated the analysis of these deletion variants and generated an additional construct that lacks both regions. Consistent with previous observations [33], deletion of region *a* had a minimal impact on *trans-*splicing, and deletion of region *b* had no effect (Fig. 2C and Supplementary Fig. S3A). In contrast, simultaneous deletion of regions *a* and *b* completely abolished *trans*-splicing. To preclude the possibility that this resulted from altered spacing between the splice donor site and region *c*, we replaced the deleted *a–b* interval with a similarly sized intronic fragment derived from the *ATPsynC* gene. This substitution failed to restore *trans*-splicing, indicating that regions *a* and *b* contained partially redundant *cis-*elements required for efficient *trans*-splicing.

To determine whether the function of the *a–b* region depends on its localization within intron 4, we transferred this combined fragment into intron 3 of the *grip* gene within the Act:*grip*_i4 construct driven by the *Act5C* promoter (Fig. 2D and Supplementary Fig. S3B). The process completely abolished *trans*-splicing, indicating that the *a–b* region must be positioned within intron 4 to support *trans*-splicing.

To further investigate the function of the *a–b* region, we generated a derivative of the M:*mod*_i4 donor in which the *a–b* region was inverted. This inversion was designed to disrupt *cis-*elements recognized at the RNA level while preserving the underlying DNA sequence and potential DNA-binding motifs. Inversion of the *a–b* region completely suppressed *trans*-splicing (Fig. 2C), indicating that this region likely contained sequence-specific RNA elements required for *trans*-splicing.

Because region *c* is also essential for efficient *trans*-splicing, we next tested whether its activity was preserved upon inversion (Fig. 2D and Supplementary Fig. S3B). Using a previously characterized donor construct lacking region *c* as a reference, we observed a similarly strong reduction in *trans*-splicing for both variants. The results indicated that inversion inactivates the function of region *c*. To exclude the possibility that independent inversion of regions *ab* and *c* disrupts their relative arrangement rather than the *cis-*elements themselves, we generated a donor construct in which the entire *abc* was inverted. This modification also completely abolished *trans*-splicing, indicating that the proximal region of intron 4 contained orientation-dependent RNA *cis-*elements required for *trans*-splicing.

### Testing the role of complementary sequences in *trans*-splicing of the *mod(mdg4)* gene

**Figure 3.**
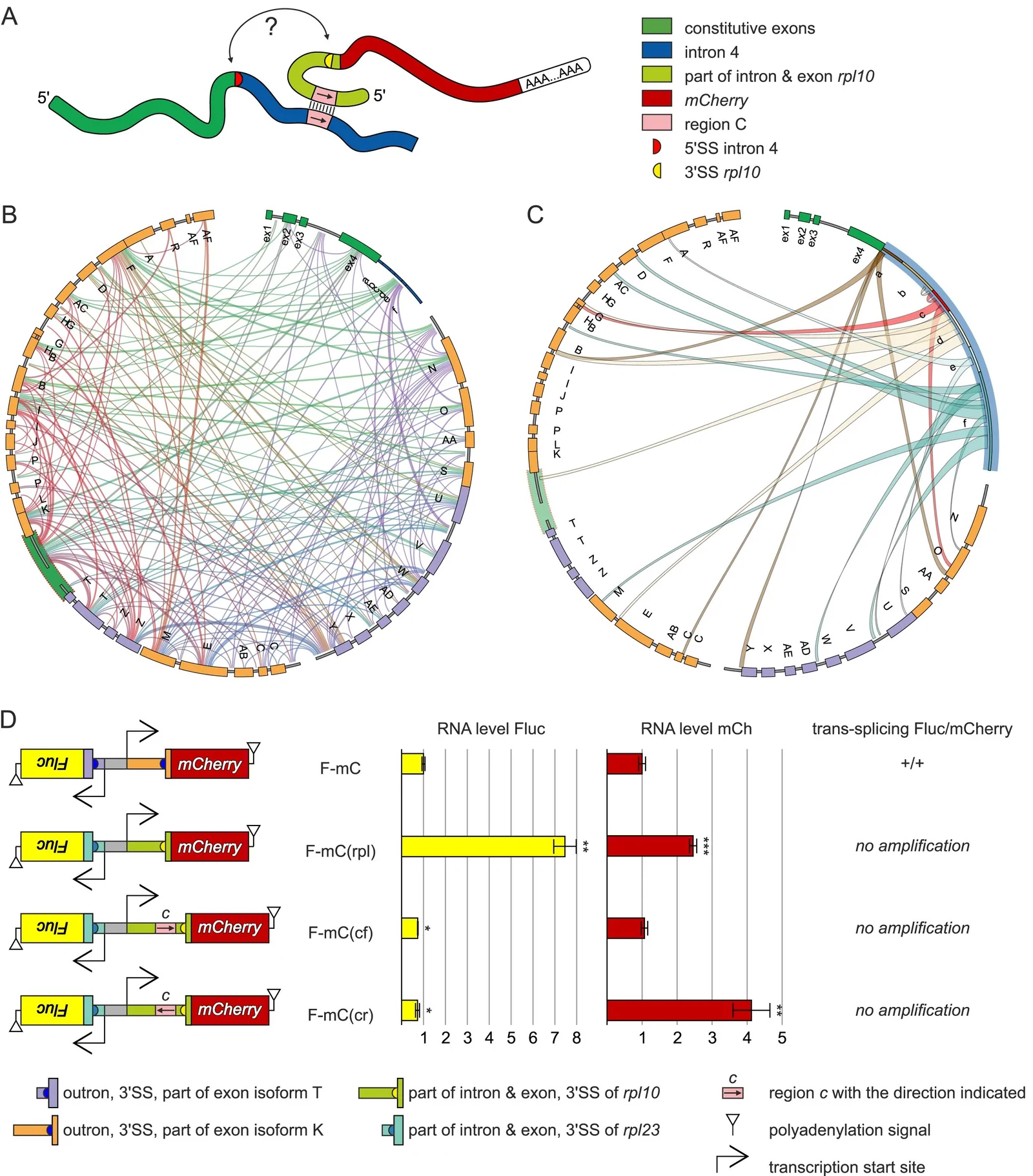
Analysis of potential complementary RNA interactions in *mod(mdg4) trans*-splicing. (**A**) Schematic representation of a potential intermolecular RNA duplex formed between donor and acceptor pre-mRNAs. Base pairing between complementary sequences may promote spatial proximity of the transcripts and facilitate *trans*-splicing. (**B**) CIRCOS diagram showing predicted intermolecular RNA duplexes within the *mod(mdg4)* locus. Only interactions with duplex lengths ≥ 25 nt and predicted free energy Δ*G* < −10 kcal/mol are shown. The thickness of lines is proportional to duplex length. For clarity, lines denoting interactions are connected to the centers of locus features and do not indicate the precise positions of predicted duplexes. (**C**) CIRCOS diagram showing predicted RNA duplexes between the proximal region of intron 4 and other transcribed regions of the *mod(mdg4)* locus. Ribbons represent interactions. For visualization purposes, intron 4 is shown enlarged, and ribbon positions correspond to the predicted locations of intermolecular RNA duplexes. (**D**) Functional analysis of the roles of outron sequences and complementary RNA pairing in *trans*-splicing. Schematics of the acceptor constructs are shown on the left. Relative transcript levels and *trans*-splicing efficiencies are shown in the center and on the right, respectively. The *c*-region was inserted in either forward or reverse orientation as indicated by the arrows within the pink boxes. Error bars represent standard deviations from three biological replicates. Statistical significance was assessed relative to the corresponding control: *P < 0.05, **P < 0.01, ***P < 0.001.

According to the widely accepted model of *trans*-splicing, pairing between complementary sequences in distinct pre-mRNAs promotes splice-site proximity and facilitates *trans*-splicing (Fig. 3A) [20, 21, 24]. Previous analyses of potential RNA–RNA interactions within the *mod(mdg4)* locus were performed using BLAST-based searches [34] and local pairwise sequence alignment [33]. However, these approaches would not identify significant complementary interactions. Importantly, neither analysis considered the contribution of intramolecular RNA secondary structures, which can substantially alter nucleotide accessibility and the spatial organization of potential interaction sites.

To address this limitation, we reanalyzed potential *trans*-interactions between RNAs transcribed from the *mod(mdg4)* locus using RNA secondary structure-aware predictions (Fig. 3B). The entire transcribed region of the *mod(mdg4)* locus was subdivided into 300 nt fragments using a sliding window with a 50 nt step size. Pairwise interactions among all fragments were then predicted using RNAcofold [53], which models intermolecular duplex formation while accounting for intramolecular secondary structures and nucleotide accessibility. Candidate interactions were selected based on low predicted Δ*G* values and reproducibility across different window positions.

The analysis identified multiple regions with the potential to mediate intermolecular RNA pairing. However, the majority of predicted interactions were localized within the clusters of alternative 3′ exons (Fig. 3B). Only a small fraction of the predicted complementary sequences were mapped within the proximal 600 bp of the fourth intron, and most of these were confined to region *d*, which previously showed minimal contribution to *trans*-splicing efficiency [33]. Notably, only a single interaction was predicted between the donor and acceptor transgenes of our model system, corresponding to pairing between region *d* of the donor intron and the K outron of the acceptor construct. However, deletion of region *d* did not impair *trans*-splicing [33]. In addition, the T outron–*Fluc* acceptor construct, for which no stable interactions with intron 4 were predicted, remained competent for *trans*-splicing. Taken together, these observations argue against a major role for complementary RNA pairing in promoting *trans*-splicing at the *mod(mdg4)* locus.

We next tested whether acceptor-derived sequences were required for *trans*-splicing. To this end, we generated an F-mC(rpl) acceptor transgene, in which the outron regions extending from the transcription start sites to the acceptor splice sites, together with short fragments of the T and K exons, were replaced with sequences of an equivalent length derived from the 3′ regions of the *rpl10* and *rpl23* introns, respectively (Fig. 3D). These heterologous intronic fragments retained the canonical 3′ splicing signals. These substitutions did not reduce transcription from the bidirectional promoter; instead, transcript levels increased. Nevertheless, despite the presence of the native bidirectional promoter and intact 3′ splice acceptor signals, *trans*-splicing was completely abolished for both acceptor variants. The results suggested that sequences located within the native outron regions upstream of the 3′ exon clusters provide essential determinants required for *trans*-splicing.

We next asked whether sequences from region *c* of intron 4 could restore *trans*-splicing in the F-mC(rpl) construct. To test this hypothesis, we generated two derivative acceptor transgenes in which region *c* was inserted into the *rpl10*-derived sequence either in the forward orientation (F-mC(cf)), preserving the native arrangement of the *cis-*elements, or in the reverse orientation (F-mC(cr)), enabling potential base pairing between complementary region *c* sequences in the donor and acceptor pre-mRNAs (Fig. 3D). However, none of these constructs restored *trans*-splicing activity. Together, the results do not support a model in which complementary pairing between donor and acceptor pre-mRNAs is the primary determinant of *trans*-splicing of the *mod(mdg4)* locus. Instead, *trans*-splicing efficiency appears to depend on specific orientation-dependent *cis-*elements within the donor intron and, in addition, sequence determinants located within the native acceptor outron regions.

### *Trans*-splicing is stimulated by several redundant sequences in the 117-bp region *c*

**Figure 4.**
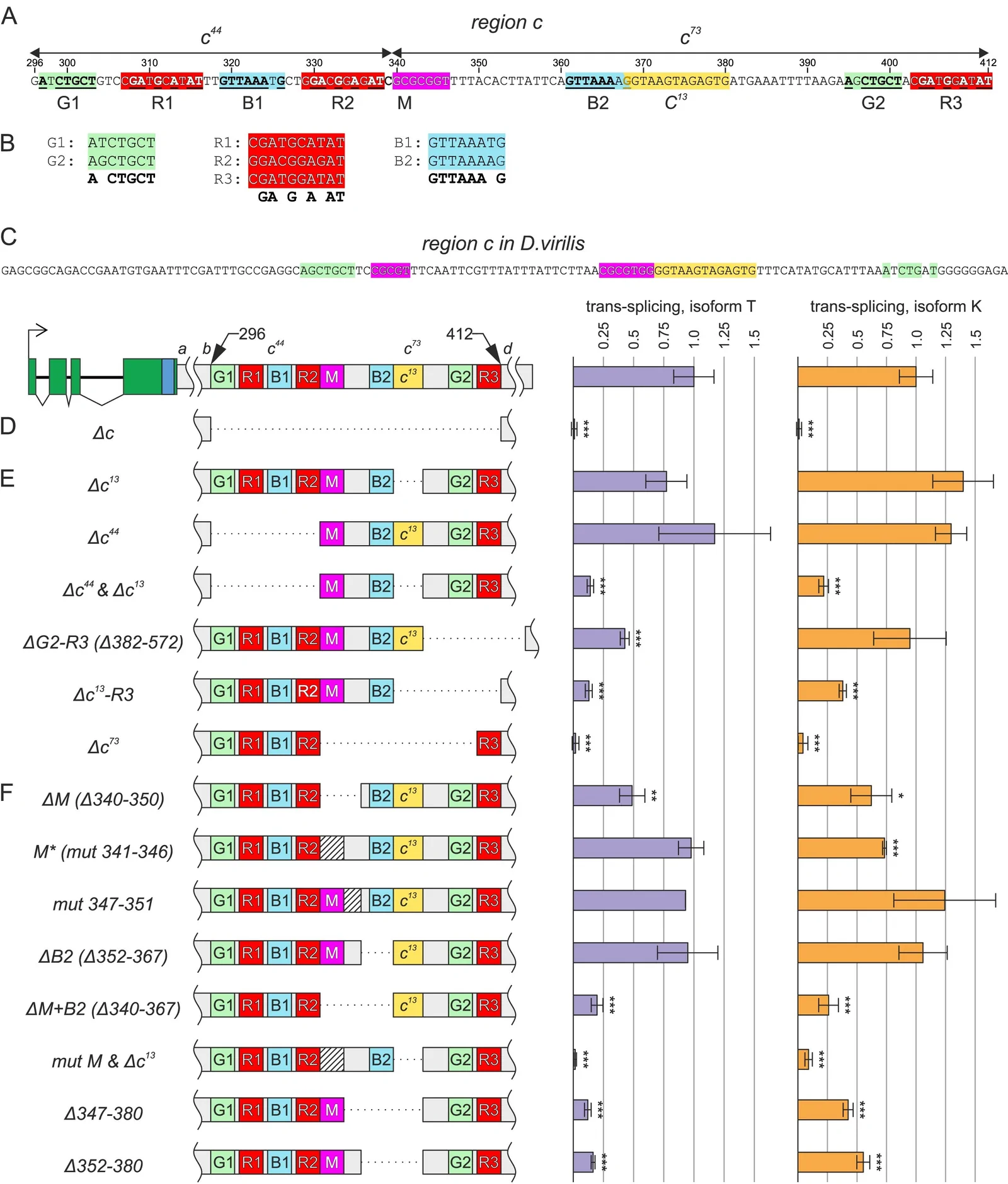
Identification of potentially functional motifs within the *c* region and their role in *trans-*splicing. (**A**) Sequence organization of the 117 bp *c* region, subdivided into the *c^44^* and *c^73^* fragments. Partially repeated motifs are highlighted in green, red, and blue. The conserved *c^13^* motif among Drosophilidae is shown in yellow. A duplicated motif identified in *D. virilis* is indicated in magenta. (**B**) Alignment of repeated sequence motifs identified within region *c*. Consensus sequences are shown below. (**C**) The sequence of the region homologous to *c* from *Drosophila virilis*. Conserved motifs shared with *D. melanogaster* are highlighted using the same color scheme as in panel (A). (**D–F**) Functional analysis of mutations within region *c*. Schematics of donor constructs are shown on the left, and the corresponding *trans*-splicing efficiencies for the T and K isoforms are shown on the right. All constructs were derived from the donor transgene containing the *mod(mdg4)* promoter and constitutive gene region (green). The colored boxes correspond to the motifs shown in panel (A–C). Dashed lines indicate deletions, and nucleotide substitutions are indicated by diagonal hatching. Each *trans*-heterozygous combination was analyzed in at least three biological replicates. Error bars represent standard deviations (*n* = 3–13). Statistical significance was assessed relative to the corresponding control: *P < 0.05, **P < 0.01, ***P < 0.001.

Previous studies [33] established that a 117-bp region (designated as the “*c* region,” located at positions 296–412 bp relative to the 5′ end of intron 4; see Fig. 4A) is critical for *trans*-splicing. Within this region lies a highly conserved 13-bp motif (*c^13^*) that is complementary to U1 snRNA [34]. However, deletion of *c^13^* had minimal impact on *trans*-splicing in a model system and at the endogenous locus [33], implying that other crucial motifs must reside within the *c* region.

To identify these elements, we conducted a detailed sequence analysis of the *c* region utilizing the MEME Suite software [56]. The analysis revealed three potential repetitive motifs (Fig. 4A and B). Although the *E*-values for these motifs exceeded 0.05, we considered their potential biological significance based on conservation and context. The motifs were subsequently color-coded and designated as G (green), R (red), and B (blue). Given our prior finding that the *D. virilis* intron supports *trans*-splicing in our model system [57], we conducted a sequence analysis of this orthologous intron, and identified both a conserved *c^13^* motif and a G motif (Fig. 4C). Additionally, we identified a novel duplicated CG-rich motif that we designated M (magenta). Notably, a similar M motif was identified in *D. melanogaster* near *c^13^*, suggesting potential functional conservation across *Drosophila* species.

To evaluate the importance of these motifs within the *c* region, we utilized a genetic model system based on crossing flies carrying donor transgenes with those carrying acceptor transgenes (Fig. 2A). In accordance with our previous findings, deletion of the entire *c* region almost completely abolished *trans*-splicing in this model system (Fig. 4D).

In our first series of experiments, we investigated the roles in *trans*-splicing of the proximal *c^44^* (G1R1B1R2) and distal G2R3 sequences from the *c* region (296–412 bp) (Fig. 4E, and Supplementary Figs S4 and S5). Deletion of the proximal *c^44^* region (Δ*c^44^*), which encompasses motifs G1, R1, B1, and R2, had no discernible effect on *trans*-splicing efficiency. Secondary structure modeling indicated that sequences within the *c^44^* region could form a stable RNA hairpin (Fig. 1B), which, together with individual motifs, might contribute to *trans*-splicing. Conversely, deletion of the region spanning 382–572 bp (which includes motifs G2 and R3) led to an approximately 50% reduction in *trans*-splicing efficiency for isoform T, without significantly affecting isoform K.

While our model system confirmed that the deletion of the *c^13^* motif alone had only a minimal effect on *trans*-splicing (Fig. 4E), we aimed to investigate the potential additive effects arising from its simultaneous deletion with other segments of the 117-bp *c* region. The simultaneous deletion of *c^13^* and *c^44^*, or *c^13^* and G2R3, resulted in a severe reduction in *trans*-splicing (Fig. 4F). Thus, *c^44^, c^13^*, and G2R3 appear to function cooperatively to enhance the efficiency of *trans*-splicing.

Our subsequent investigation focused on the roles of individual segments within the 340–367-bp (M-B2) region (Fig. 4F and Supplementary Fig. S4). Neither individual deletion nor mutation of these components significantly affected *trans*-splicing. However, the most substantial decrease was observed following the deletion of a segment containing the M motif (a motif conserved between *D. virilis* and *D. melanogaster)* combined with its adjacent sequences (Δ340–350). These findings collectively supported a model wherein multiple motifs within the 340–367-bp (M + B) region function cooperatively. Furthermore, mutation of the M motif in combination with the deletion of *c^13^* led to an almost complete inactivation of *trans*-splicing.

Taken together, these findings demonstrate an unexpectedly high degree of redundancy and cooperation among the diverse motifs in this region that collectively act to promote efficient *trans*-splicing.

### Confirmation of interchangeability and redundancy of motifs in *trans-*splicing at the endogenous *mod(mdg4)* locus

The model system based on donor and acceptor transgenes integrated into the 22A genomic locus indicated that a cluster of motifs within the *c* region plays an important role in mediating *trans*-splicing.

**Figure 5.**
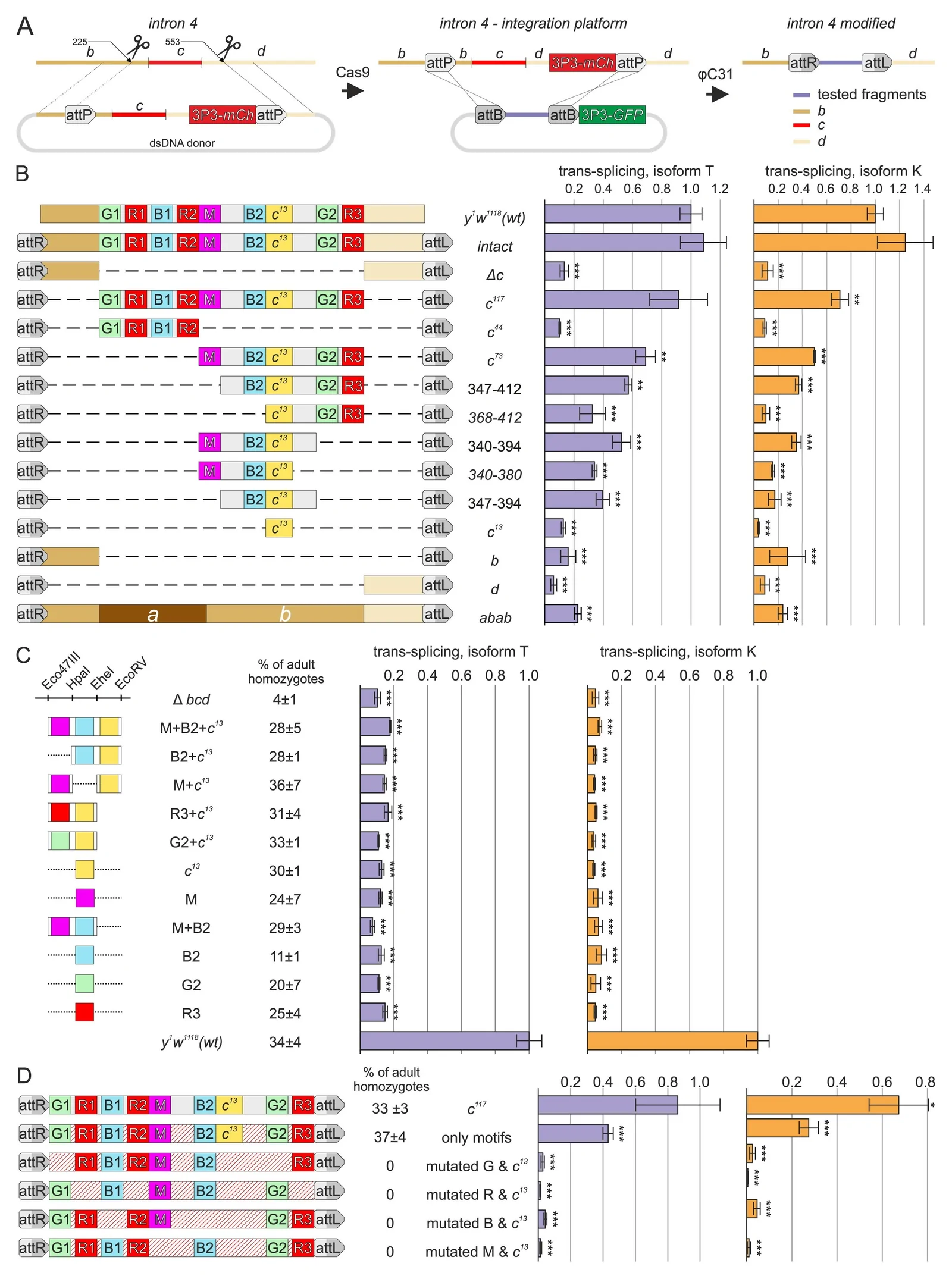
Functional dissection of *cis-*elements required for *trans*-splicing at the endogenous *mod(mdg4)* locus. (**A**) The strategy employed in genome engineering of the fourth intron. Cas9 cleavage sites within regions *b* and *d* are indicated by the scissors. The donor repair plasmid contains homologous arms (dashed lines) flanking the intact *c* region together with two attP integration sites. Successful integration events were identified using the *3xP3-mCherry* marker cassette. Subsequently, φC31-mediated recombination was used to introduce modified intronic fragments flanked by attB sites, thereby replacing the marker cassette. The *3xP3-eGFP* marker was used to confirm integration of the plasmid backbone and was not incorporated into the tested alleles. (**B**) Functional analysis of distinct *c*-region fragments. Schematics of tested intronic variants are shown on the left. Putative motifs are represented by colored rectangles, and deleted regions are indicated by dashed lines. Relative *trans*-splicing efficiencies for the T and K isoforms are shown on the right. (**C**) Functional analysis of various combinations of conserved motifs. The percentage of viable homozygous adults is indicated in the center. Relative *trans*-splicing efficiencies for the T and K isoforms are shown on the right. (**D**) The effect of spacer-sequence mutations between conserved motifs on *trans*-splicing efficiency. Putative motifs are shown as colored rectangles, whereas mutated regions are indicated by diagonal hatching. *Trans*-splicing efficiencies for the T and K isoforms measured in pupae are shown on the right. Each line was analyzed in at least three biological replicates. Error bars represent standard deviations (*n* = 3–18). Statistical significance was assessed relative to the corresponding control: *P < 0.05, **P < 0.01, ***P < 0.001.

To substantiate these findings at the endogenous *mod(mdg4)* locus, we engineered a landing platform for targeted integration of various segments of intron 4 (*mod(mdg4)^attPx2^*). This platform involved replacing the native sequence spanning 226–553 bp of the fourth intron with the same *mod(mdg4)* sequences along with an *mCherry* reporter gene; both were flanked by two attP recombination sites (Fig. 5A). The viability of homozygous *mod(mdg4)^attPx2^* flies confirmed that this genomic modification did not disrupt essential biological functions. Using genetic crosses, we first established a homozygous *mod(mdg4)^attPx2^* line that also carried the transgene expressing the *φC31* integrase under the control of the nanos promoter (*y^1^w^1118^ P(y[+t7.7]=nanos-phiC31\int.NLS)X; mod(mdg4)^attPx2^*). The integrated sequences were flanked by attB sites. The *eGFP* reporter gene was inserted outside the attB sites, marking the plasmid backbone and enabling the selection of successful transformants that did not express *eGFP* or *mCherry*. Plasmids containing the test sequences, along with a control plasmid carrying the intact DNA fragment spanning the 226–553-bp positions, were then injected into *mod(mdg4)^attPx2^* embryos.

We initiated the experiment by substituting the 226–553 bp region with a linker sequence comprising only a MCS, thus generating the *mod(mdg4)^Δbcd^*. This modification led to a pronounced decrease in fly viability. Approximately 90% of homozygous flies died during the pupal stage, with the remaining few survivors being sterile and dying within days. Considering that null *mod(mdg4)* mutants typically display lethality during metamorphosis [58, 59], the *mod(mdg4)^Δbcd^* mutant likely exhibits considerably diminished expression of its isoforms. This outcome highlights the indispensable role of this genomic region for the proper functioning of the *mod(mdg4)* locus.

Our subsequent experiment aimed to determine whether the intact intron fragment (226–553 bp), the *c^117^* segment (296–412 bp), and its subregions *c^44^* (296–339 bp) and *c^73^* (340–412 bp) could restore *trans-*splicing and thereby restore fly viability (Fig. 5B and Supplementary Figs S6, S9A, and S11, and Supplementary Table S1). In contrast to the lethal effects observed in homozygous *mod(mdg4)^Δbcd^* flies, the integration of each of these tested DNA fragments restored the viability of the *mod(mdg4)* mutants (Supplementary Fig. S11 and Supplementary Table S1).

*Trans-*splicing efficiency was quantified by extracting total RNA from 2- to 3-day-old males, followed by RT-qPCR. The assay utilized primers designed to span the *trans-*spliced junctions, with the forward primer annealed to the fourth exon of the donor and the reverse primer targeting one of the alternative exons (T, K, or Z). The probes were specifically designed to cover the junction between the donor and acceptor exons. All results were normalized against the expression of the housekeeping genes *vha100-1* and *CG9067*. For comparative analysis, *trans-*splicing efficiency for isoforms T, K, and Z was set relative to the control line *y^1^w^1118^*, which was defined as 1.0.

Restoration of the intact *mod(mdg4)* intronic sequence (a 226–553 bp fragment) resulted in *trans*-splicing levels comparable to those observed in the control *y^1^w^1118^* line (Fig. 5B and Supplementary Fig. S9A). This result demonstrated that neither the genomic editing of the *mod(mdg4)* intron nor the retention of *attL/R* sites within the *b* and *d* regions compromised *trans*-splicing efficiency. Conversely, introduction of only the *c^117^* sequence resulted in a slight reduction in *trans*-splicing relative to the control, likely due to the partial deletions of the adjacent *b* and *d* regions in this construct. Insertion of the *b* (226–296 bp) or *b + d* (226–296 + 413–553 bp) DNA fragments into *mod(mdg4)Δbcd* flies rescued their viability and partially restored *trans*-splicing efficiency (Fig. 5B).

When evaluated separately, the *c^117^* subregions–the *c^44^* (the proximal portion) and *c^73^* (the distal portion)–yielded distinct results. The *c^44^* fragment exhibited weak induction of *trans*-splicing, whereas *c^73^* sustained *trans*-splicing at a level only slightly below that of *c^117^*. Notably, *c^73^* contains the *c^13^* and M motifs, both of which were implicated in promoting *trans*-splicing in our model system. We then generated several truncated derivatives of the *c^73^* segment to identify the critical determinants of *trans*-splicing within this region (Fig. 5B). In the 347–412 bp and 368–412 bp DNA fragments, sequential deletion of the M and B2 motifs from the proximal side of the c*^73^* region led to a progressive reduction in *trans*-splicing, consistent with a functional role for both motifs. Similarly, in the 340–394 bp and 340–380 bp DNA fragments, the G2–R3 and spacer sequences were sequentially removed from the distal end of the *c^73^* region, and this also correlated with a stepwise decrease in *trans*-splicing efficiency. While the *c^13^* motif alone induced relatively low *trans*-splicing activity, the *c^13^* motif flanked by the B2 motif and spacer sequences (the 347–394 bp fragment) resulted in a relatively high level of *trans*-splicing. Taken together, these results suggest that the intervening spacer sequences also contribute to *trans*-splicing efficiency. The role of these spacer elements is further supported by a twofold increase in *trans*-splicing induced by the native 340–380 bp (Fig. 5B) fragment compared to an artificial analog containing only the M, B2, and *c^13^* motifs (M + B2 + *c^13^*) (Fig. 5C). It is likely that the functional motifs are wider than predicted, that strict requirements for precise inter-motif spacing exist, or that additional essential motifs reside within these spacers. Therefore, the results collectively suggest that both the exact length of the *trans*-splicing-determining motifs and their relative genomic positions are critical to their functioning.

We also tested whether duplication of the upstream a–b region could compensate for the 226–553 bp deletion and restore *trans*-splicing by inserting the *a *+ *b* (11–295 bp) DNA fragment into the *mod(mdg4)attPx2* platform (Fig. 5B). Duplication of the *a *+ *b* region only weakly restored *trans*-splicing relative to the *b* (226–295 bp) control. This result suggests that efficient *trans*-splicing requires a diverse combination of motifs for different RBPs, rather than the simple duplication of a specific subset of these elements.

### Motifs and spacer sequences are essential for *trans*-splicing

Since the *c*^13^ motif alone yielded very low *trans*-splicing activity, we asked whether the addition of another motif, such as M, R3, B2, or G2, could enhance *trans*-splicing efficiency (Fig. 5C and Supplementary Fig. S7). We utilized the *mod(mdg4)^attPx2^* platform to integrate *c*^13^ in combination with each individual motif. Despite the demonstrated role of these elements in promoting *trans*-splicing, their pairwise combination with *c*^13^ did not lead to a marked increase in *trans*-splicing efficiency. This observation further supported the hypothesis that the intervening sequences between motifs are essential for their functional activity.

Although the isolated *c*^13^ motif only marginally increased *trans*-splicing when integrated into the *mod(mdg4)^attPx2^* platform, it completely restored mutant viability compared to the *Δbcd* line (Fig. 5C, and Supplementary S11 and Supplementary Table S1). Thus, even minor increases in *trans*-splicing efficiency are critical for the sufficient expression of Mod(mdg4) isoforms necessary for survival. We then tested whether other individual motifs integrated into the *mod(mdg4)^attPx2^* platform could rescue viability similarly to *c*^13^. We found that all tested motifs (M, B2, G2, and R3) only weakly restored *trans*-splicing efficiency, mirroring the effect of *c*^13^ alone (Fig. 5C). Notably, M, G2, and R3 partially restored mutant viability, albeit less effectively than *c*^13^, whereas integration of the *B2* motif resulted in only a slight increase in survival. Interestingly, the combined integration of *M* and *B2* almost completely rescued the viability of the resulting mutants.

These results demonstrate that the *G2, R3, M*, and *B2* motifs are actively involved in driving *trans*-splicing, corroborating the conclusions derived from the transgenic model system (see Fig. 4). Collectively, a distinct group of motifs within the fourth intron contributes to *trans*-splicing efficiency, with varying degrees of impact: the *G* and *R* motifs have minor roles, whereas the *M* and *c*^13^ motifs provide the most substantial contributions. Remarkably, the absence of any single motif has only a minimal impact on *trans*-splicing efficiency, further underscoring their functional redundancy.

To further of the functions of the sequences between motifs, derivatives of the *c^117^* fragment were integrated into the *mod(mdg4)^attPx2^* platform. These included a variant in which all nucleotides located between the motifs underwent replacement, effectively retaining only the motifs themselves (designated as *mod(mdg4)^om^*, only motifs), and another variant that combined substitutions of these inter-motif nucleotides with specific mutations within the *c^13^* and M motifs (*mod(mdg4)^mutM&c13^*) (Fig. 5D, and Supplementary Fig. S8 and S9C). Substitution of the nucleotides between motifs within the *mod(mdg4)^om^* line resulted in a considerable reduction in *trans-*splicing efficiency relative to the *y^1^w^1118^* control line. Surprisingly, homozygous *mod(mdg4)^mutM&c13^* flies died during the pupal stage. Thus, insertion of the mutant DNA fragment at the attP site results in lethality of pupae compared to the pattern observed in the *mod(mdg4)* null mutants [58, 59]. Accordingly, *trans-*splicing was detected only at a rudimentary level in *mod(mdg4)^mutM&c13^* flies.

Taken together, the results indicate that the spacers between the identified motifs play an active role in *trans*-splicing, likely contributing to the core motifs recognized by RBPs.

### Identification of proteins interacting with the proximal region of the last common intron

We performed *in vitro* RNA affinity purification followed by mass spectrometry to identify RBPs associated with the proximal 412 bp region of the fourth intron. We conducted *in vitro* transcription of an RNA fragment termed *abc* that contained the last 20 nucleotides of exon 4 together with intronic regions *a, b*, and *c*. As a control, we generated a second RNA fragment, termed *cba*, in which the sequence spanning nucleotides 11–412 of the intron was inverted. This inversion corresponds to the *abc^inv^* transgene previously analyzed *in vivo* (Fig. 2D), where *trans*-splicing was completely abolished. The RNAs were biotinylated at the 3′ end and used for pull-down of RNA-associated protein complexes on streptavidin-coated magnetic beads.

**Figure 6.**
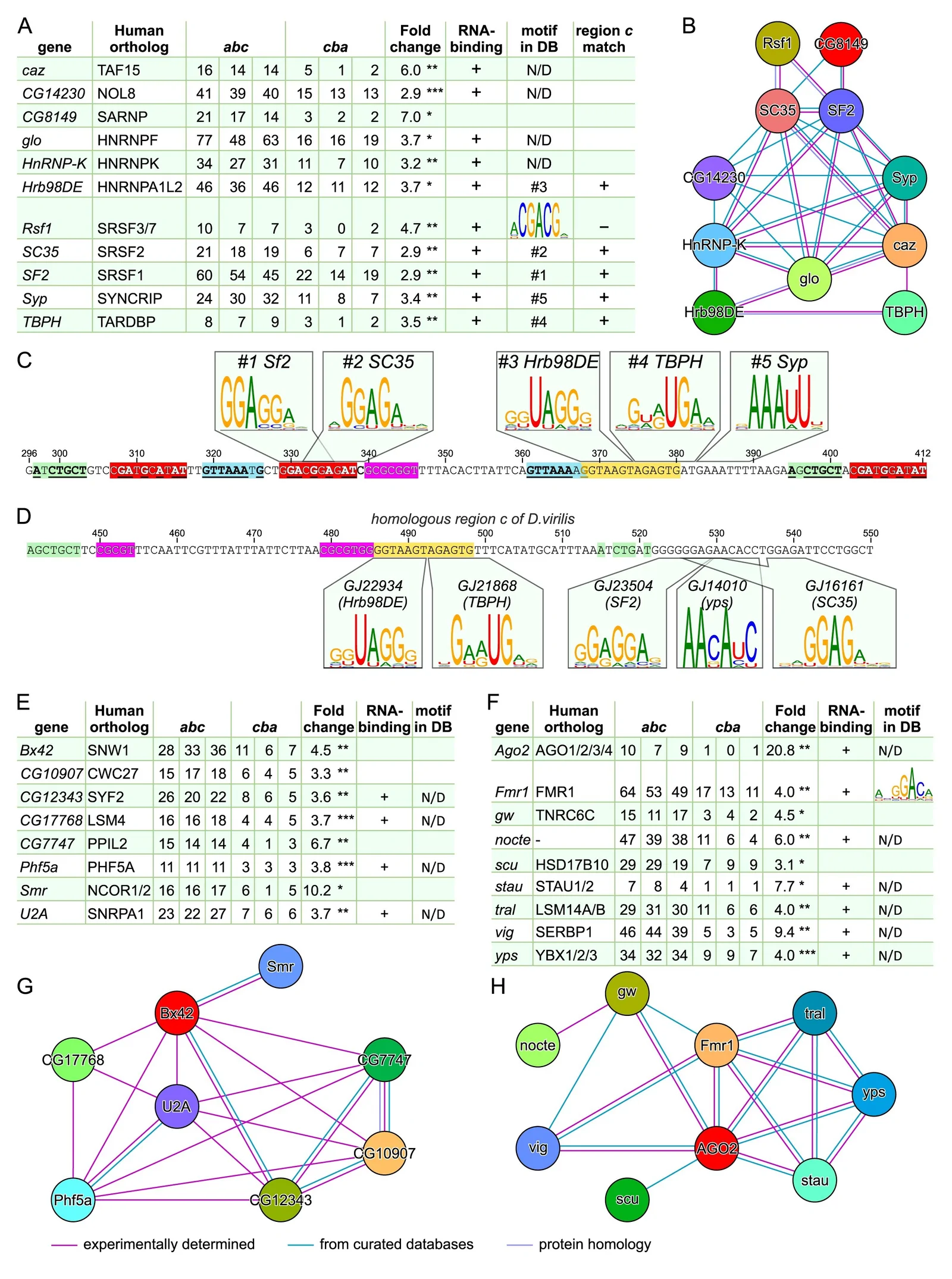
Identification of the protein complexes associated with the proximal region of intron 4. (**A, E**, and **F**) Proteins enriched following *in vitro* RNA affinity purification coupled with mass spectrometry using the *abc* RNA fragment or the inverted *cba* control RNA. The *abc* RNA contained the proximal intronic regions *abc* in the native orientation, whereas the control *cba* RNA carried the same sequence in the inverted orientation corresponding to the *abc^inv^* construct. The tables show normalized count values obtained from three independent experiments, the average fold enrichment, the annotated RNA-binding activity, the availability of experimentally determined RNA-binding motifs, and the presence of predicted binding sites within the *c^117^* region. Statistical significance of protein enrichment relative to the control sample: *P < 0.05, **P < 0.01, ***P < 0.001. (**B, G**, and **H**) Interaction networks of proteins enriched on the *abc* RNA fragment based on annotated interactions from the STRING [60] and FlyBase [36] databases. Nodes represent proteins identified by affinity purification, and edges indicate experimentally supported or annotated functional interactions. (**C**) The distribution of predicted binding motifs for proteins from the first RNA-binding subcomplex across the *c^117^* region of *D. melanogaster*. Binding sites were identified using experimentally determined RNA-binding motifs available for *D. melanogaster* proteins [61]. Conserved and repeated sequence motifs shown in Fig. 4 are indicated using the same color scheme. (**D**) The distribution of predicted binding motifs within the homologous *c^117^* region of *D. virilis*. Binding sites were predicted using RNA-binding motif models available for the corresponding *D. virilis* orthologs [61]. Conserved sequence motifs are shown using the same color coding as in (C).

RNA-coated magnetic beads were incubated with a nuclear extract from S2 cells. RNA-associated proteins were identified by label-free quantitative mass spectrometry (MS). After filtering (see Materials and methods), 188 proteins (Supplementary Table S4) were retained for further analysis. A protein interaction analysis using the STRING database [60] identified three groups of proteins with annotated functional interactions, suggesting the presence of distinct RNA-associated protein complexes (Fig. 6).

The most prominent subgroup comprised 11 proteins, 10 of which possessed annotated RNA-binding activity (Fig. 6A and B). RNA-binding motifs have been experimentally characterized for six proteins in this group [61, 62]. Notably, the motifs for Hrb98DE and TBPH, which directly interact, were located adjacent to one another within the *c^13^* element (Fig. 6C). The motif for Syp is positioned near *c^13^* within a spacer region that also contributes to *trans*-splicing efficiency (Fig. 5B). The SC35 motif matched the R2 sequence, while a similar motif recognized by the related splicing factor Sf2 was likewise found exclusively within R2. No binding motif for Rsf1 was identified within region *c*. Unfortunately, the binding motifs of the remaining four RBPs in this subgroup are unknown.

We also compared the distribution of predicted RNA-binding motifs between *D. virilis* and *D. melanogaster*. As expected, orthologs of Hrb98DE and TBPH were predicted to bind the conserved *c^13^* region. The predicted binding sites for the *D. virilis* orthologs of Sf2 and SC35 were shifted downstream of *c^13^* relative to their positions in *D. melanogaster*. Notably, SC35 may potentially bind two adjacent sites in the *D. virilis* sequence. Although no Syp-binding site was detected in *D. virilis*, a predicted binding site for Yps, detected among the enriched proteins, was identified adjacent to the Sf2 and SC35 motifs.

A second subgroup corresponded to components of the U2 snRNP-associated complex, although only four proteins in this group were annotated as RNA-binding (Fig. 6E and G). Because binding motifs for these proteins have not yet been determined, their direct interaction with the intronic RNA cannot currently be assessed.

Finally, we identified a third subgroup consisting of proteins associated with RNA interference effector complexes [63], including Ago2, Vig, and dFXR/Fmr1 (Fig. 6F and H). A known binding motif for Fmr1 was identified within region *b* of the *mod(mdg4)* intron. However, the binding motifs of the other RBPs in this group remain unknown.

Taken together, these analyses identified three interacting protein subnetworks enriched in the proximal region of intron 4. Notably, one protein complex showed a strong association with the intronic region functionally required for *trans*-splicing.

### Impaired *trans*-splicing reduces mod(mdg4) mRNA isoforms but spares Mod(mdg4)-67.2 protein abundance and function

**Figure 7.**
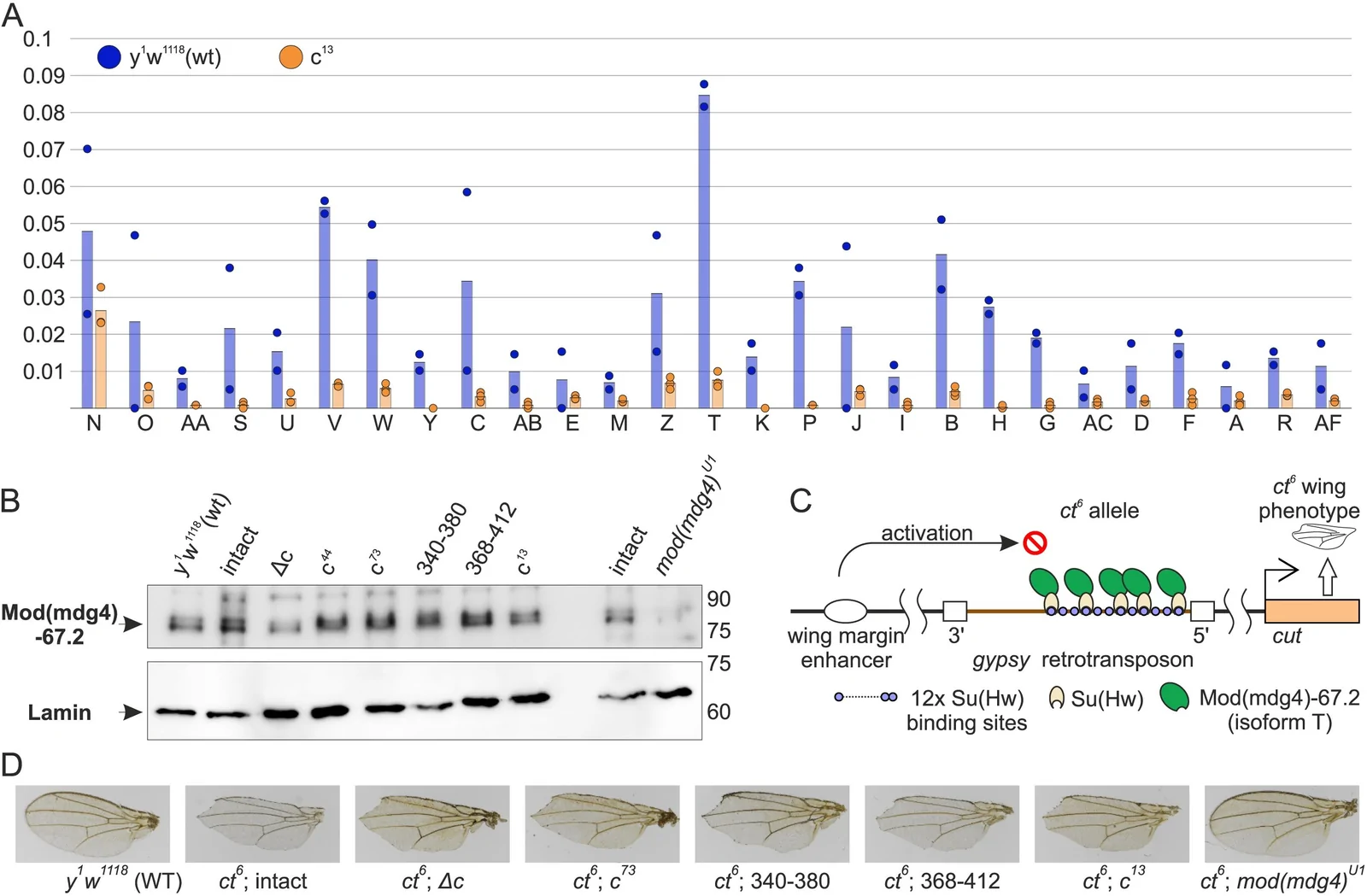
Reduced *trans*-splicing efficiency does not impair Mod(mdg4)-67.2 protein function. (**A**) RNA-seq analysis of the *trans*-splicing efficiency of *mod(mdg4)* isoforms in 2- and 3-day-old adult males from the *c^13^* mutant and the *y^1^w^1118^* control line. For each isoform, the number of reads spanning the *trans*-splice junction was normalized to the number of reads spanning the exon 3–4 splice junction within the constitutive region of *mod(mdg4)*. The bars represent mean values, and dots indicate individual biological replicates (*n* = 2 for *y^1^w^1118^* and *n* = 3 for *c^13^*). (**B**) Western blot analysis of Mod(mdg4)-67.2 protein levels in flies carrying mutations within intron 4. Protein extracts from 2- and 3-day-old adult males were probed with antibodies specific to the Mod(mdg4)-67.2 isoform. Anti-lamin antibodies were used as a loading control. The *mod(mdg4)^u1^* mutant, which lacks expression of Mod(mdg4)-67.2, was used as a negative control. (**C**) Schematic representation of the functional assay for Mod(mdg4)-67.2 activity based on the *ct^6^* allele. The *gypsy* retrotransposon inserted between the wing-margin enhancer and the *cut* promoter contains Su(Hw)-binding sites that recruit the Su(Hw)/Mod(mdg4)-67.2 insulator complex, thereby blocking enhancer–promoter communication and producing the characteristic cut-wing phenotype. Loss of Mod(mdg4)-67.2 function disrupts insulator activity and restores the wild-type wing phenotype. (**D**) Representative wing phenotypes of *ct^6^* males homozygous for the indicated *mod(mdg4)* alleles. Mutations affecting *trans*-splicing efficiency retain the characteristic cut-wing phenotype, whereas the *mod(mdg4)^u1^* mutation restores the wild-type wing morphology.

The *c^13^* flies were fully viable and displayed a normal phenotype despite a more-than-10-fold reduction in *trans*-splicing efficiency for the T, K, and Z isoforms. The lack of an observable functional consequence despite such a substantial decrease in mRNA encoding these protein isoforms remained unclear. One plausible explanation would be that the observed reduction in *trans-*splicing impacts only a subset of the 3′ alternative exons. Consequently, to explore this hypothesis, we conducted RNA sequencing on *mod(mdg4)* gene-enriched RNA samples derived from the *mod(mdg4)^c13^* and control *y^1^w^1118^* lines. To quantify *trans-*splicing levels, we compared the number of reads spanning alternative *trans-*splice junctions in both lines, normalized against reads spanning the constitutive exon 3–exon 4 splice junction (Fig. 7A). Our analysis revealed that all isoforms exhibited considerably reduced *trans-*splicing efficiency in the *mod(mdg4)^c13^* line. Simultaneously, there was an approximately 2-fold increase in the splicing efficiency of 3′ exons encoding the N and O isoforms. This latter phenomenon was most likely a consequence of *cis*-splicing becoming active, a pathway typically suppressed at the native *mod(mdg4)* locus [33].

An alternative hypothesis to account for the viability of *mod(mdg4)^c13^* flies posits that normal isoform expression may occur during early developmental stages, with reduced levels appearing exclusively in adult flies. To examine this hypothesis, we quantified the expression levels of the T, K, and Z isoforms throughout the larval and pupal stages of *Drosophila* development (Supplementary Fig. S10). Our analysis revealed a significant decrease in *trans-*splicing at both developmental stages, demonstrating that the reduction in *trans-*splicing is not restricted to adult flies.

Our investigation into the correlation between synthesized mRNA levels and their corresponding protein products focused on the Mod(mdg4)^T^ isoform, also known as Mod(mdg4)-67.2. This specific isoform interacts with the architectural C2H2 zinc-finger protein Su(Hw), forming an insulator complex that blocks enhancer–promoter communication [30]. The *mod(mdg4)^u1^* mutation, in which the Mod(mdg4)-67.2 isoform is not expressed, was used as a negative control. We conducted a western blot analysis to quantify Mod(mdg4)-67.2 protein levels in the imago from the control lines *y^1^w^1118^* and *mod(mdg4)^intact^*, generated by restoring the deleted intronic sequences in the *mod(mdg4)^attPx2^* background and from the mutant lines *mod(mdg4)^c44^, mod(mdg4)^c73^, mod(mdg4)^340–380^, mod(mdg4)^368–412^, mod(mdg4)^c13^*, and *mod(mdg4)^u1^*.

Protein extracts prepared from 2- to 3-day-old adult males were probed with antibodies specific to Mod(mdg4)-67.2 (Fig. 7B). As expected, the Mod(mdg4)-67.2 protein was undetectable in *mod(mdg4)^u1^* extracts, whereas there was a clear signal in the control line, confirming antibody specificity. Despite the observed considerable reduction in *mod(mdg4)^T^* mRNA *trans-*splicing efficiency, Mod(mdg4)-67.2 protein levels were comparable among the tested controls and all mutant variants. This observation indicates that the marked reduction in *trans*-spliced mRNA is not reflected in a corresponding decrease in protein abundance.

We employed a sensitive functional assay based on the *ct^6^* mutation to gain further insight into Mod(mdg4)-67.2. The *ct^6^* allele arises from the insertion of the *gypsy* mobile element into the 85-kb region separating the wing margin enhancer from the *cut* promoter [28, 64]. The Su(Hw)/Mod(mdg4)-67.2 complex binds to this *gypsy* insulator, consequently inhibiting wing margin enhancer activity and producing the characteristic serrated wing phenotype (Fig. 7C). When *ct^6^* was combined with homozygous *mod(mdg4)^u1^* mutants, which are deficient in Mod(mdg4)-67.2, the wild-type wing blade phenotype was almost completely restored due to loss of enhancer blocking.

In stark contrast, *ct^6^* flies carrying all evaluated *mod(mdg4)* mutations consistently displayed the characteristic *cut* wing phenotype, confirming robust enhancer blocking mediated by the *gypsy* insulator (Fig. 7D). Thus, Mod(mdg4)-67.2 protein abundance and function are maintained despite an approximately 10-fold reduction in functional *mod(mdg4)^T^* mRNA.

## Discussion

The *mod(mdg4)* locus represents a unique model for investigating the mechanisms of *trans*-splicing, as all *mod(mdg4)* mRNAs are generated through *trans*-splicing between a pre-mRNA encoding the common N-terminal region and one of 31 alternative exons specifying distinct C-terminal domains. In *Drosophila*, as in other metazoans, *trans*-splicing is generally suppressed due to its potential to generate aberrant chimeric mRNAs encoding deleterious proteins. Canonical *cis-*splicing is therefore tightly coupled to transcription and occurs predominantly co-transcriptionally, thereby ensuring accurate and efficient mRNA maturation [1, 65, 66]. Although some *cis-*splicing events occur post-transcriptionally and contribute to developmental and stress-responsive regulation of gene expression [67], polyadenylated transcripts that retain introns typically remain associated with chromatin until splicing is completed. In this context, the *mod(mdg4)* locus represents an exceptional example of extensive reprogramming of the canonical pre-mRNA processing pathway.

Our results indicate that the proximal 600 bp region of intron 4 is sufficient to redirect pre-mRNA processing toward *trans*-splicing independent of the promoter and upstream constitutive sequences. We found no convincing evidence supporting a model in which *trans*-splicing is driven by stable RNA duplex formation between the regulatory region of intron 4 and acceptor pre-mRNAs. Computational analyses identified only limited, functionally unsupported complementary interactions, while the introduction of an artificial complementary interaction failed to restore *trans*-splicing. These findings argue against a major role for intermolecular RNA pairing in the *mod(mdg4)* locus and instead support a distinct mechanism that joins 5′ and 3′ exons transcribed from separate promoters.

Our analyses revealed a high density of partially redundant RBP motifs distributed throughout the *c^117^* region (296–412 bp). At the same time, efficient *trans*-splicing depends on currently unidentified elements located within regions *a* (11–108 bp) and *b* (109–295 bp). Region *d* contributes more modestly, but its functional importance becomes apparent upon disruption of motifs within region *c*. This extensive redundancy likely explains why deleting individual motifs or small groups of motifs has only limited effects on *trans*-splicing efficiency, whereas progressively removing multiple motifs produces cumulative defects. Importantly, attempts to isolate individual core motifs were insufficient to sustain efficient *trans*-splicing. Taken together, these observations suggest that *trans*-splicing depends not on a single determinant sequence but on the cooperative action of multiple *cis-*elements distributed across the proximal intronic region.

Using *in vitro* RNA affinity purification, we identified several protein groups that were specifically enriched on the native *abc* RNA fragment compared with the inverted control sequence. The most compelling candidates are proteins forming interaction-connected subnetworks that likely assemble on the proximal 412 bp intronic region and directly participate in *trans*-splicing induction. Particularly notable is the first RNA-binding complex, in which five proteins possess predicted binding sites within the *c^117^* region. The Hrb98DE/TBPH heterodimer binds adjacent motifs within the conserved *c^13^* element, which is critical for efficient *trans*-splicing. Two additional proteins in this complex, SC35 and Sf2, recognize overlapping motifs within the R2 region but not within the related R1 or R3 repeats, suggesting functional specialization among partially homologous sequence elements. In addition, Syp recognizes a motif immediately downstream of *c^13^* that also contributes to *trans*-splicing. The *c^117^* region likely contains additional recognition sites for cooperatively interacting RBPs. Some proteins enriched by RNA affinity purification but lacking characterized RNA-binding specificity may recognize motifs located within regions *a, b*, and *c^117^*.

Previous studies have demonstrated that closely spaced RBP motifs can cooperatively regulate splicing through multivalent interactions among RBPs bound to pre-mRNAs [68–71]. Biophysical studies further showed that multivalent RBP–RNA interactions can drive the formation of dynamic condensates through liquid–liquid phase separation (LLPS) [72–74]. Such condensates can increase local molecular concentrations by orders of magnitude relative to the surrounding nucleoplasm, thereby stabilizing assembly on RNAs containing multiple clustered binding motifs. We propose that a similar mechanism operates at the *mod(mdg4)* locus, where multiple interacting RBPs recognize densely packed intronic motifs and assemble into a specialized RNP compartment that alters canonical *cis-*splicing and transcriptional progression. Collective recruitment of these factors may induce currently uncharacterized modifications of both the spliceosome and the RNA pol II complex.

**Figure 8.**
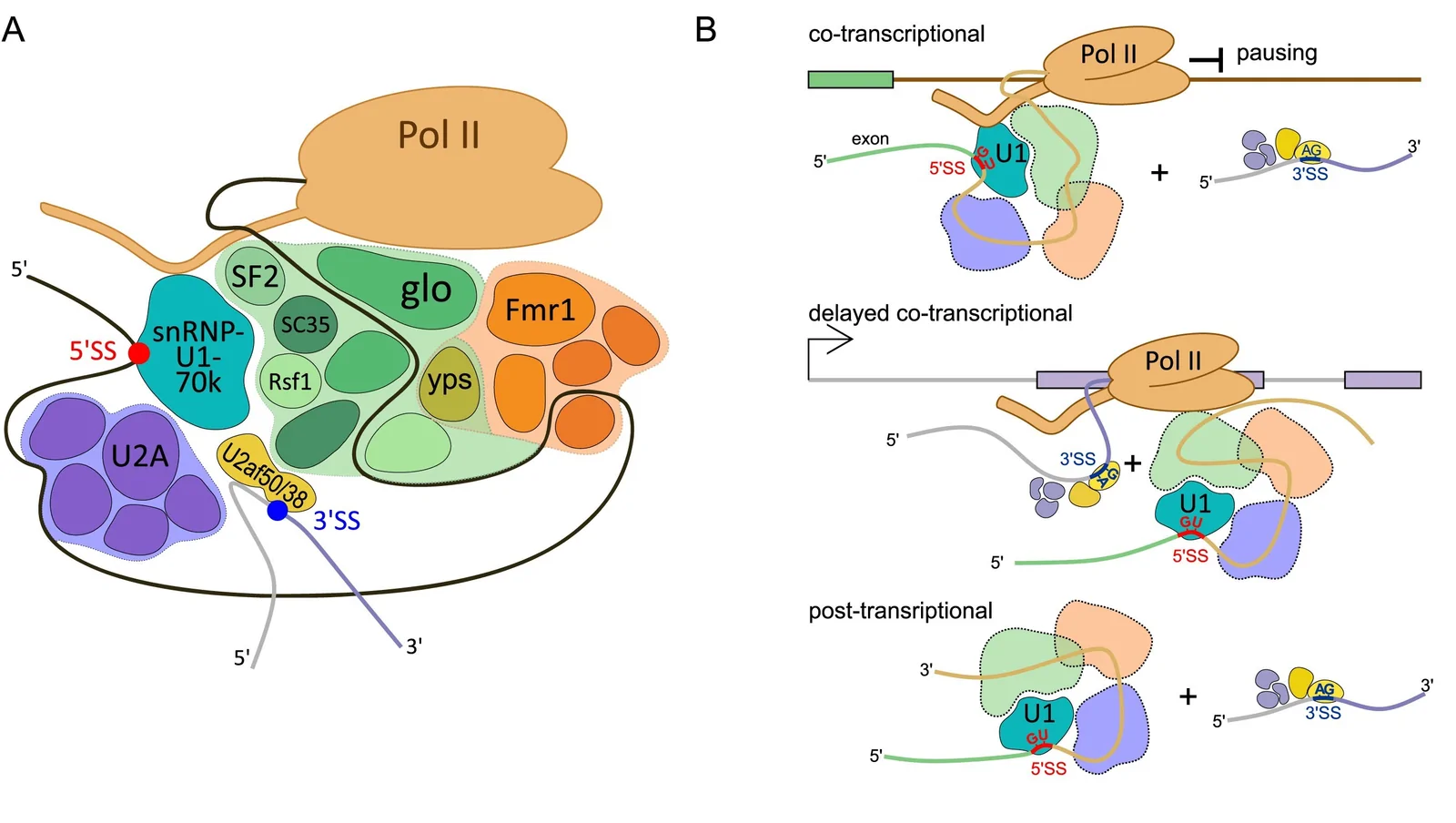
Several proposed models of protein complex assembly and *trans*-splicing at the *mod(mdg4)* locus. (**A**) Schematic representation of interactions between RNA pol II, spliceosomal components, and protein complexes identified on the proximal region of intron 4. Proteins belonging to the first RNA-binding subcomplex are shown in green; components of the U2-associated complex are shown in purple, and proteins associated with RNA interference-related complexes are shown in orange. Core spliceosomal factors interacting with the fourth intron, including U1 snRNP-70K and U2AF38/50, are highlighted in cyan and yellow, respectively. (**B**) Proposed models of *trans*-splicing following the assembly of RNA–protein complexes on intron 4. In the co-transcriptional model, the donor transcript remains associated with its transcription site until it interacts with acceptor pre-mRNAs. In the delayed co-transcriptional model, the donor transcript, together with associated protein complexes, relocates to the transcription site of acceptor transcripts before *trans*-splicing occurs. In the post-transcriptional model, donor and acceptor pre-mRNAs interact after the transcript is released from the chromatin to generate mature *trans*-spliced mRNAs.

The identified protein complexes establish extensive connections with one another, as well as with components of the transcriptional machinery and the spliceosome (Fig. 8A). Within the first RNA-binding complex, Glo may provide a link to the transcriptional machinery, as its human homolog, HNRNPF, physically associates with the RNA pol II holoenzyme complex [75]. Glo is also connected to Fmr1, a component of the third complex [76], potentially linking transcriptional and RNA-associated pathways. The human homolog of Yps, YBX1, interacts with homologs of proteins from both the first and third complexes [77], suggesting a possible connection between these protein assemblies. The first complex is further linked to U1 snRNP through interactions involving U1-70K, SC35, Sf2, and Rsf1. Additional components may interact with the U2AF50/U2AF38 heterodimer, which is involved in 3′ splice-site recognition. Another spliceosomal component, U2A, is incorporated into the second extended complex. Together, the proteins associated with the intron and their putative connections to spliceosomal and transcriptional components suggest that a large ribonucleoprotein assembly may form on the proximal region of the nascent intron 4. The composition and spatial organization of this assembly could alter the canonical transcriptional and splicing program, thereby promoting *trans*-splicing.

At least three mechanistic scenarios can be envisioned as occurring following initiation of the *trans*-splicing program (Fig. 8B). In the co-transcriptional *trans*-splicing model, the donor transcript, together with RNA pol II and associated RNP complexes, remains near the donor transcription site after transcriptional pausing or termination until it encounters an acceptor pre-mRNA. In this scenario, specificity may be determined by protein factors associated with acceptor outrons and by spatial proximity between donor and acceptor transcription sites. Thus, donor splicing remains coupled to transcription, whereas acceptor processing occurs after the transcript is released from the chromatin. In the delayed co-transcriptional model, RNA pol II, together with the donor pre-mRNA and associated RNP complexes, relocates to promoters, driving transcription of the 3′ exon clusters. Alternatively, donor-associated RNP complexes are recruited to assembling acceptor transcription complexes. *Trans*-splicing would then occur during transcription of the alternative exons and would therefore resemble conventional co-transcriptional alternative splicing at the acceptor locus. Finally, in the post-transcriptional model, *trans*-splicing between donor and acceptor pre-mRNA occurs only after the transcript is released into the interchromosomal space of the nucleus. Further research is required to determine which of these models operates *in vivo*.

The extensive redundancy of RNA motifs within the proximal region of intron 4 likely contributes to the efficiency and robustness of *trans*-splicing. Notably, deletions reducing *trans*-splicing efficiency by approximately one order of magnitude did not substantially alter Mod(mdg4)-67.2 protein levels or its biological activity. These findings therefore indicate that a compensatory mechanism maintains Mod(mdg4)-67.2 protein abundance and function despite markedly reduced *trans*-splicing efficiency. The molecular basis of this compensation remains unknown. One possibility is that elevated translational efficiency originally evolved to compensate for inefficient ancestral *trans*-splicing, with subsequent evolutionary optimization of *trans*-splicing reducing the selective pressure for stringent translational control. Consistent with this idea, several proteins identified within the Ago2-associated complex, including Vig [78], Fmr1 [76], and GW182 [79], function as translational repressors. One hypothetical scenario is that the Ago2-containing complex simultaneously promotes *trans*-splicing while suppressing translation of the resulting mRNAs. If so, disruption of intronic motifs could reduce recruitment of this complex and partially compensate for reduced *trans*-splicing by increasing translation of *mod(mdg4)* mRNAs.

Our results suggest that *trans*-splicing may have evolved through the gradual accumulation of intronic motifs recognized by RBPs that normally participate in canonical *cis-*splicing. Although the *mod(mdg4)* locus generates all isoforms exclusively through *trans*-splicing, many other loci combine *cis-* and *trans*-splicing to produce alternative mRNAs. The *lola* locus represents a prominent example in which numerous isoforms are generated through mixed *cis/trans*-splicing mechanisms [80, 81]. Moreover, analysis of *D. melanogaster/D. sechellia* hybrids identified *trans*-splicing events at approximately 80 genes [81], suggesting that this mechanism is more widespread than previously thought.

In other metazoans, *trans*-splicing likely provides an efficient strategy for generating extensive repertoires of alternative mRNAs and protein isoforms from complex loci [25, 82]. The silkworm *mod(mdg4)* locus illustrates this concept, encoding >200 distinct protein isoforms [57, 83]. Because *trans*-splicing appears to depend primarily on combinatorial arrays of intronic RNA motifs rather than on specialized dedicated machinery, similar mechanisms may operate broadly across the animal kingdom. However, rapid evolutionary turnover of RBP-binding motifs likely generates substantial species-specific variation in *trans*-splicing-inducing sequences. Consistent with this idea, intronic sequences from *D. virilis* remain capable of inducing *trans*-splicing in *D. melanogaster*, whereas the corresponding silkworm sequences do not [57].

*Trans*-splicing substantially expands the coding and regulatory capacity of complex loci by enabling combinatorial assembly of alternative mRNAs from independently transcribed modules. Because this mechanism can coexist with canonical *cis-*splicing and appears to rely primarily on distributed intronic regulatory motifs, *trans*-splicing may be considerably more prevalent than currently believed. Defining its full biological significance, however, will require elucidation of the molecular architecture and dynamics of the RNP assemblies that initiate and coordinate *trans*-splicing.

A limitation of this study is the lack of direct experimental validation of the role of the identified RBPs in *trans*-splicing. Given the high redundancy among motifs recognized by different RBPs, inactivating a single RBP is unlikely to disrupt *trans*-splicing. Therefore, dissecting of the individual contributions of these proteins will probably require either combined inactivation of multiple RBPs or the use of a sensitized genetic background, both of which represent a promising avenue for future research.

## Supplementary Material

gkag823_Supplemental_Files

## Data Availability

NGS data have been deposited at the NCBI Gene Expression Omnibus (GEO) under accession number GSE302152. The mass spectrometry proteomics data have been deposited to the ProteomeXchange Consortium via the PRIDE partner repository with the dataset identifier PXD079014 and 10.6019/PXD079014.
