# Supplementary material for: Multiple intron motifs have redundant functions in the trans-splicing of the mod(mdg4) locus
Source: Nucleic Acids Res. 2026 Aug 20;54(15):gkag823. doi: 10.1093/nar/gkag823 (PMC13490949; doi:10.1093/nar/gkag823)
Supplement: Supplementary file 1 [file gkag823_supplemental_files.zip › 140826110147_suplementary.pdf]

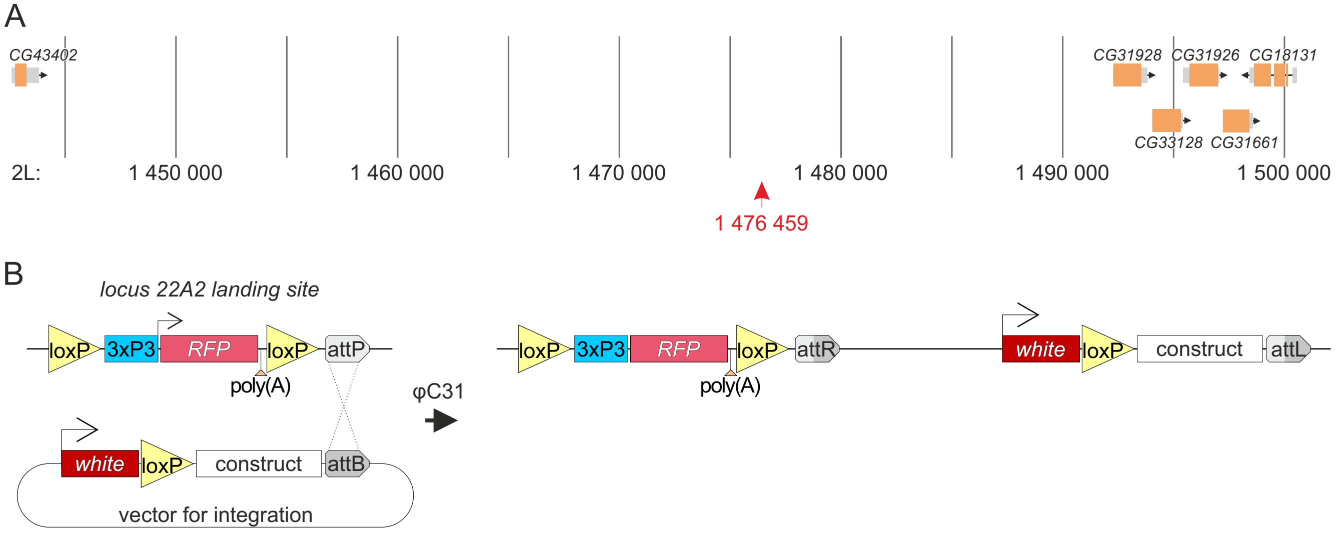

Supplementary Figure S1. **(A)** Genomic map of the 22A locus. The red arrow marks the integration site. Orange-colored neighboring genes lie over 15 kb away. Coordinates correspond to chromosome 2L nucleotide numbering. **(B)** Schematic of donor and acceptor model construct integration into the 22A locus. The platform line is equipped with an attP docking site and an *RFP* reporter, driven by the 3xP3 promoter, at the 22A2 locus. Additionally, the φC31 integrase gene, controlled by the germline-specific vasa promoter, is integrated on the X chromosome. Integration vectors contain donor or acceptor sequences, a white reporter, and an attB site. Site-specific recombination between attP and attB sites results in genomic integration of the entire plasmid. Successful events were identified by pigmented eyes.

Reproduced from Beginyazova et. al. (40) under the Creative Commons Attribution (CC BY) license.

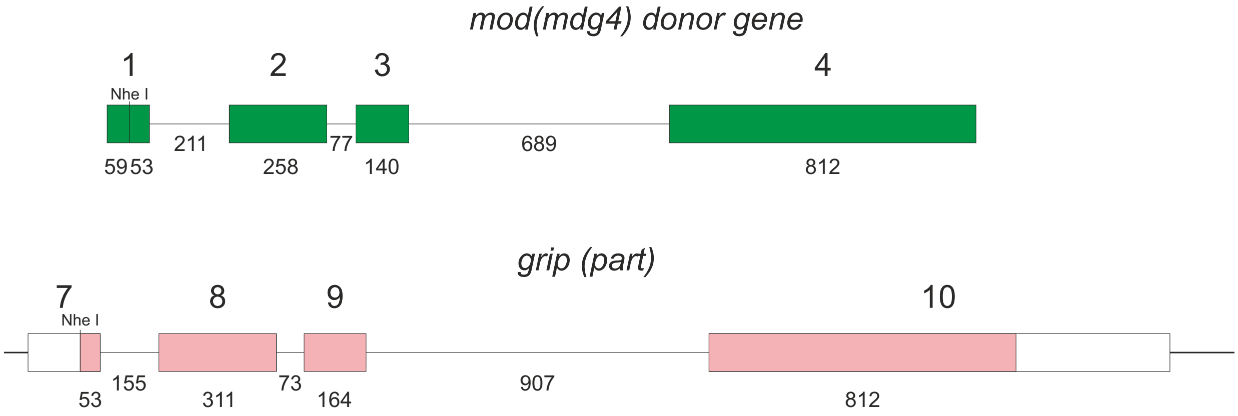

Supplementary Figure S2. Scaled diagram of a *grip* gene fragment (pink) used to replace sequences of the *mod(mdg4)* gene (green). Exon and intron lengths are indicated below the schematic, and exon numbering is relative to the gene start and shown above.

Reproduced from Beginyazova et. al. (40) under the Creative Commons Attribution (CC BY) license.

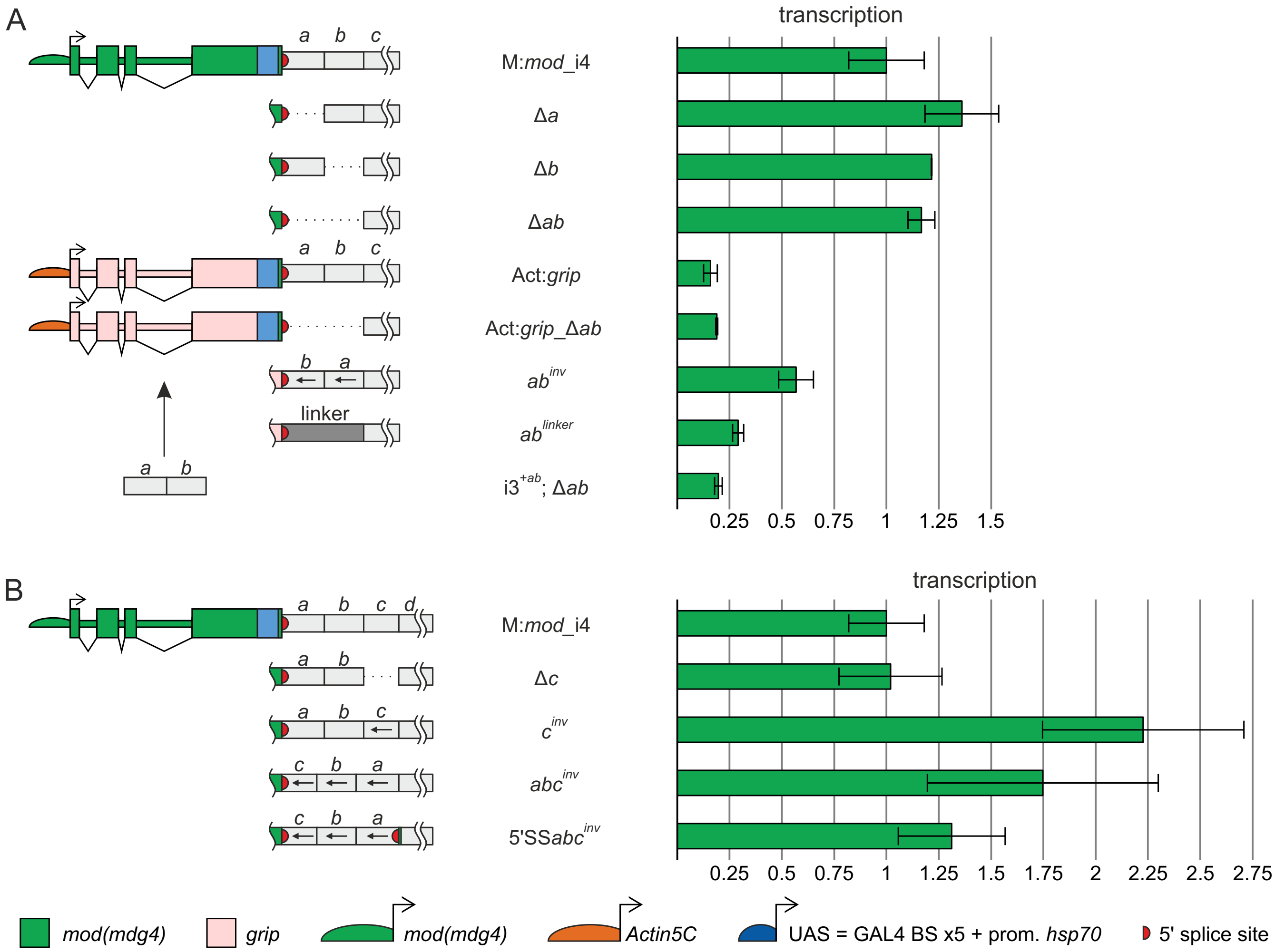

Supplementary Figure S3. Functional analysis of the proximal region of intron 4 in *mod(mdg4)* trans-splicing. (A) Effects of deletions and inversions within regions *a* and *b* on steady-state level of the donor transcript. Schematics of donor constructs are shown on the left. The donor segment is driven by either the endogenous *mod(mdg4)* promoter (green), the *Act5C* promoter (orange), or the UAS promoter (blue), and contains gene-body sequences derived from either *mod(mdg4)* (green) or *grip* (pink). The splice donor site is indicated by a red semicircle. Deletions are shown as dashed lines and inversions as black arrows. The dark gray segment corresponds to a control intronic fragment derived from *ATPsynC*. The construct labeled "i3^{+ab}; Δab" contains the a–b region inserted into intron 3 of the *grip* gene derivative. Construct names are centrally located. Relative level of the donor transcriptare shown on the right. (B) Effects of inversion of region *c* or the entire abc fragment on trans-splicing efficiency. Construct organization and graphical annotations are as described in (A).

```
                            *       300         *       320         *       340
intron       : AAAGAGCACTAGGCAGATCTGCTGTCCGATGCATATTTGTTAAATGCTGGACGGAGATCG
Δc           : AAAGAGCACTAGGCAGATC-----------------------------------------
Δc44         : AAAGAGCACTAGGCAG--------------------------------------------
Δ382-572     : AAAGAGCACTAGGCAGATCTGCTGTCCGATGCATATTTGTTAAATGCTGGACGGAGATCG
Δ340-367     : AAAGAGCACTAGGCAGATCTGCTGTCCGATGCATATTTGTTAAATGCTGGACGGAGATC-
Δ340-350     : AAAGAGCACTAGGCAGATCTGCTGTCCGATGCATATTTGTTAAATGCTGGACGGAGATC-
mut 341-346  : AAAGAGCACTAGGCAGATCTGCTGTCCGATGCATATTTGTTAAATGCTGGACGGAGATCG
mut 347-351  : AAAGAGCACTAGGCAGATCTGCTGTCCGATGCATATTTGTTAAATGCTGGACGGAGATCG
Δ352-367     : AAAGAGCACTAGGCAGATCTGCTGTCCGATGCATATTTGTTAAATGCTGGACGGAGATCG

                            *       360         *       380         *       400
intron       : CGCGGTTTTACACTTATTCAGTTAAAAGGTAAGTAGAGTGATGAAATTTTAAGAAGCTGC
Δc           : ------------------------------------------------------------
Δc44         : CGCGGTTTTACACTTATTCAGTTAAAAGGTAAGTAGAGTGATGAAATTTTAAGAAGCTGC
Δ382-572     : CGCGGTTTTACACTTATTCAGTTAAAAGGTAAGTAGAGTGA-------------------
Δ340-367     : ---------------------------GGTAAGTAGAGTGATGAAATTTTAAGAAGCTGC
Δ340-350     : ----------CACTTATTCAGTTAAAAGGTAAGTAGAGTGATGAAATTTTAAGAAGCTGC
mut 341-346  : AATTCATTTACACTTATTCAGTTAAAAGGTAAGTAGAGTGATGAAATTTTAAGAAGCTGC
mut 347-351  : CGCGGTAAATGACTTATTCAGTTAAAAGGTAAGTAGAGTGATGAAATTTTAAGAAGCTGC
Δ352-367     : CGCGGTTTTAC----------------GGTAAGTAGAGTGATGAAATTTTAAGAAGCTGC

                            *       420         *       440         *       460
intron       : TACGATGGATATCTGATGTCAACGTGAAGCCGAACCCGTGAAGAGCTGCAACTACTCAAG
Δc           : ----------ATCTGATGTCAACGTGAAGCCGAACCCGTGAAGAGCTGCAACTACTCAAG
Δc44         : TACGATGGATATCTGATGTCAACGTGAAGCCGAACCCGTGAAGAGCTGCAACTACTCAAG
Δ382-572     : ------------------------------------------------------------
Δ340-367     : TACGATGGATATCTGATGTCAACGTGAAGCCGAACCCGTGAAGAGCTGCAACTACTCAAG
Δ340-350     : TACGATGGATATCTGATGTCAACGTGAAGCCGAACCCGTGAAGAGCTGCAACTACTCAAG
mut 341-346  : TACGATGGATATCTGATGTCAACGTGAAGCCGAACCCGTGAAGAGCTGCAACTACTCAAG
mut 347-351  : TACGATGGATATCTGATGTCAACGTGAAGCCGAACCCGTGAAGAGCTGCAACTACTCAAG
Δ352-367     : TACGATGGATATCTGATGTCAACGTGAAGCCGAACCCGTGAAGAGCTGCAACTACTCAAG

                            *       480         *       500         *       520
intron       : GTAATACCGGCACTCTTAAAACCACACCTATCCATATATTTTTCCAAAATTTGCAGGCAT
Δc           : GTAATACCGGCACTCTTAAAACCACACCTATCCATATATTTTTCCAAAATTTGCAGGCAT
Δc44         : GTAATACCGGCACTCTTAAAACCACACCTATCCATATATTTTTCCAAAATTTGCAGGCAT
Δ382-572     : ------------------------------------------------------------
Δ340-367     : GTAATACCGGCACTCTTAAAACCACACCTATCCATATATTTTTCCAAAATTTGCAGGCAT
Δ340-350     : GTAATACCGGCACTCTTAAAACCACACCTATCCATATATTTTTCCAAAATTTGCAGGCAT
mut 341-346  : GTAATACCGGCACTCTTAAAACCACACCTATCCATATATTTTTCCAAAATTTGCAGGCAT
mut 347-351  : GTAATACCGGCACTCTTAAAACCACACCTATCCATATATTTTTCCAAAATTTGCAGGCAT
Δ352-367     : GTAATACCGGCACTCTTAAAACCACACCTATCCATATATTTTTCCAAAATTTGCAGGCAT

                            *       540         *       560         *       580
intron       : TTGTAATTGTTTATTTGACTGAGCAAAGCGAGTAATTGGAGTTTTCAATCTTACATGATC
Δc           : TTGTAATTGTTTATTTGACTGAGCAAAGCGAGTAATTGGAGTTTTCAATCTTACATGATC
Δc44         : TTGTAATTGTTTATTTGACTGAGCAAAGCGAGTAATTGGAGTTTTCAATCTTACATGATC
Δ382-572     : ----------------------------------------------------ACATGATC
Δ340-367     : TTGTAATTGTTTATTTGACTGAGCAAAGCGAGTAATTGGAGTTTTCAATCTTACATGATC
Δ340-350     : TTGTAATTGTTTATTTGACTGAGCAAAGCGAGTAATTGGAGTTTTCAATCTTACATGATC
mut 341-346  : TTGTAATTGTTTATTTGACTGAGCAAAGCGAGTAATTGGAGTTTTCAATCTTACATGATC
mut 347-351  : TTGTAATTGTTTATTTGACTGAGCAAAGCGAGTAATTGGAGTTTTCAATCTTACATGATC
Δ352-367     : TTGTAATTGTTTATTTGACTGAGCAAAGCGAGTAATTGGAGTTTTCAATCTTACATGATC
```

Supplementary Figure S4. Multiple sequence alignment of intron 4 fragments, which correspond to the constructs in Fig. 4D-E. Coordinates above indicate positions relative to the intron 4 start, with every tenth nucleotide numbered or marked by an asterisk. The top sequence represents the reference genome. Dashes (–) denote deletions. Sequence coloring reflects identity levels: 100% identity is yellow, >80% is blue, and >60% is gray.

```
                                 *       300         *       320         *       340
intron          : AAAGAGCACTAGGCAGATCTGCTGTCCGATGCATATTTGTTAAATGCTGGACGGAGATCG
Δc13            : AAAGAGCACTAGGCAGATCTGCTGTCCGATGCATATTTGTTAAATGCTGGACGGAGATCG
Δc73            : AAAGAGCACTAGGCAGATCTGCTGTCCGATGCATATTTGTTAAATGCTGGACGGAGATC-
Δc44 & Δc13     : AAAGAGCACTAGGCA--------------------------------------------G
mut_M1 & Δc13   : AAAGAGCACTAGGCAGATCTGCTGTCCGATGCATATTTGTTAAATGCTGGACGGAGATCG
Δ347-c13        : AAAGAGCACTAGGCAGATCTGCTGTCCGATGCATATTTGTTAAATGCTGGACGGAGATCG
Δ352-c13        : AAAGAGCACTAGGCAGATCTGCTGTCCGATGCATATTTGTTAAATGCTGGACGGAGATCG
Δc13-412        : AAAGAGCACTAGGCAGATCTGCTGTCCGATGCATATTTGTTAAATGCTGGACGGAGATCG

                                 *       360         *       380         *       400
intron          : CGCGGTTTTACACTTATTCAGTTAAAAGGTAAGTAGAGTGATGAAATTTTAAGAAGCTGC
Δc13            : CGCGGTTTTACACTTATTCAGTTAAAA-------------ATGAAATTTTAAGAAGCTGC
Δc73            : ------------------------------------------------------------
Δc44 & Δc13     : CGCGGTTTTACACTTATTCAGTTAAAA-------------ATGAAATTTTAAGAAGCTGC
mut_M1 & Δc13   : AATTCATTTACACTTATTCAGTTAAAA-------------ATGAAATTTTAAGAAGCTGC
Δ347-c13        : CGCGGT----------------------------------ATGAAATTTTAAGAAGCTGC
Δ352-c13        : CGCGGTTTTAC-----------------------------ATGAAATTTTAAGAAGCTGC
Δc13-412        : CGCGGTTTTACACTTATTCAGTTAAAA---------------------------------

                                 *       420
intron          : TACGATGGATATCTGATGTC
Δc13            : TACGATGGATATCTGATGTC
Δc73            : ------------CTGATGTC
Δc44 & Δc13     : TACGATGGATATCTGATGTC
mut_M1 & Δc13   : TACGATGGATATCTGATGTC
Δ347-c13        : TACGATGGATATCTGATGTC
Δ352-c13        : TACGATGGATATCTGATGTC
Δc13-412        : ----------ATCTGATGTC
```

Supplementary Figure S5. This multiple sequence alignment of intron 4 fragments, which correspond to the constructs in Fig. 4F. The sequences shown are derivatives with deletions introduced concurrently with the removal of the c^{13} element. Coordinates above indicate positions relative to the intron 4 start, with every tenth nucleotide numbered or marked by an asterisk. The top row shows the reference genome intron sequence. Dashes (–) denote deletions. Sequence similarity is color-coded: 100% identity is yellow, >80% is blue, and >60% is gray.

```
          attB                                 Eco47III    EheI
                                                     HpaI
WT      : ----------------------------------------------------------CGCTGGCCATTA
intact  : GGGTGCCAGGGCGTGCCCTTGGGCTCCCCGGGCGCGTA--------------------CGCTGGCCATTA
c117    : GGGTGCCAGGGCGTGCCCTTGGGCTCCCCGGGCGCGTAGCGCT---------------------------
c44     : GGGTGCCAGGGCGTGCCCTTGGGCTCCCCGGGCGCGTAGCGCTGTTAACGGCGCCGAT------------
c73     : GGGTGCCAGGGCGTGCCCTTGGGCTCCCCGGGCGCGTAGCGCTGTTAACGGCGCCGAT------------
368-412 : GGGTGCCAGGGCGTGCCCTTGGGCTCCCCGGGCGCGTAGCGCT---------------------------
340-380 : GGGTGCCAGGGCGTGCCCTTGGGCTCCCCGGGCGCGTAGCGCT---------------------------
c13     : GGGTGCCAGGGCGTGCCCTTGGGCTCCCCGGGCGCGTAGCGCC---------------------------
Δc      : GGGTGCCAGGGCGTGCCCTTGGGCTCCCCGGGCGCGTA--------------------CGCTGGCCATTA

                                                                    c177
          240         *       260         *       280         *       300
WT      : GTTAATAAGTAGTTTACGGGCCTGTGTTCTATTTCGAATCGACAAAGAGCACTAGGCAGATCTGCTGTCC
intact  : GTTAATAAGTAGTTTACGGGCCTGTGTTCTATTTCGAATCGACAAAGAGCACTAGGCAGATCTGCTGTCC
c117    : ----------------------------------------------------------GATCTGCTGTCC
c44     : ----------------------------------------------------------GATCTGCTGTCC
c73     : ----------------------------------------------------------------------
368-412 : ----------------------------------------------------------------------
340-380 : ----------------------------------------------------------------------
c13     : ----------------------------------------------------------------------
Δc      : GTTAATAAGTAGTTTACGGGCCTGTGTTCTATTTCGAATCGACAAAGAGCACTAGGCAGATC--------

                                                                      c13
            *       320         *       340         *       360         *
WT      : GATGCATATTTGTTAAATGCTGGACGGAGATCGCGCGGTTTTACACTTATTCAGTTAAAAGGTAAGTAGA
intact  : GATGCATATTTGTTAAATGCTGGACGGAGATCGCGCGGTTTTACACTTATTCAGTTAAAAGGTAAGTAGA
c117    : GATGCATATTTGTTAAATGCTGGACGGAGATCGCGCGGTTTTACACTTATTCAGTTAAAAGGTAAGTAGA
c44     : GATGCATATTTGTTAAATGCTGGACGGAGATC--------------------------------------
c73     : --------------------------------GCGCGGTTTTACACTTATTCAGTTAAAAGGTAAGTAGA
368-412 : ------------------------------------------------------------GGTAAGTAGA
340-380 : --------------------------------GCGCGGTTTTACACTTATTCAGTTAAAAGGTAAGTAGA
c13     : ------------------------------------------------------------GGTAAGTAGA
Δc      : ----------------------------------------------------------------------

          380         *       400         *       420         *       440
WT      : GTGATGAAATTTTAAGAAGCTGCTACGATGGATATCTGATGTCAACGTGAAGCCGAACCCGTGAAGAGCT
intact  : GTGATGAAATTTTAAGAAGCTGCTACGATGGATATCTGATGTCAACGTGAAGCCGAACCCGTGAAGAGCT
c117    : GTGATGAAATTTTAAGAAGCTGCTACGATGGATAT-----------------------------------
c44     : ----------------------------------------------------------------------
c73     : GTGATGAAATTTTAAGAAGCTGCTACGATGGATAT-----------------------------------
368-412 : GTGATGAAATTTTAAGAAGCTGCTACGATGGATAT-----------------------------------
340-380 : GT--------------------------------------------------------------------
c13     : GT--------------------------------------------------------------------
Δc      : ---------------------------------ATCTGATGTCAACGTGAAGCCGAACCCGTGAAGAGCT

            *       460         *       480         *       500         *
WT      : GCAACTACTCAAGGTAATACCGGCACTCTTAAAACCACACCTATCCATATATTTTTCCAAAATTTGCAGG
intact  : GCAACTACTCAAGGTAATACCGGCACTCTTAAAACCACACCTATCCATATATTTTTCCAAAATTTGCAGG
c117    : ----------------------------------------------------------------------
c44     : ----------------------------------------------------------------------
c73     : ----------------------------------------------------------------------
368-412 : ----------------------------------------------------------------------
340-380 : ----------------------------------------------------------------------
c13     : ----------------------------------------------------------------------
Δc      : GCAACTACTCAAGGTAATACCGGCACTCTTAAAACCACACCTATCCATATATTTTTCCAAAATTTGCAGG
```

```
                                                    EcoRV attB
          520         *       540         *
WT      : CATTTGTAATTGTTTATTTGACTGAGCAAAGCGAGT----------------------------------
intact  : CATTTGTAATTGTTTATTTGACTGAGCAAAGCGAGT------GGGTGCCAGGGCGTGCCCTTGGGCTCCC
c117    : ------------------------------------GATATCGGGTGCCAGGGCGTGCCCTTGGGCTCCC
c44     : ---------------------------------------ATCGGGTGCCAGGGCGTGCCCTTGGGCTCCC
c73     : ---------------------------------------ATCGGGTGCCAGGGCGTGCCCTTGGGCTCCC
368-412 : ------------------------------------GATATCGGGTGCCAGGGCGTGCCCTTGGGCTCCC
340-380 : ------------------------------------GATATCGGGTGCCAGGGCGTGCCCTTGGGCTCCC
c13     : ------------------------------------GATATCGGGTGCCAGGGCGTGCCCTTGGGCTCCC
Δc      : CATTTGTAATTGTTTATTTGACTGAGCAAAGCGAGT------GGGTGCCAGGGCGTGCCCTTGGGCTCCC

            *
WT      : ----------
intact  : CGGGCGCGTA
c117    : CGGGCGCGTA
c44     : CGGGCGCGTA
c73     : CGGGCGCGTA
368-412 : CGGGCGCGTA
340-380 : CGGGCGCGTA
c13     : CGGGCGCGTA
Δc      : CGGGCGCGTA
```

Supplementary Figure S6. Multiple sequence alignment of intron 4 fragments used as constructs for integration into *mod(mdg4)* intron 4 (corresponding to constructs in Fig. 5B). The topmost row labels feature start sites, including attB sites, restriction sites, and *c* region fragment boundaries. The second row provides coordinates relative to the intron 4 start, with every 10 bp numbered or marked by an asterisk. The uppermost sequence (WT) shows the wild-type reference genome sequence that was replaced during editing. Dashes (–) indicate deletions. Sequence similarity is color-coded: >77% identity is yellow, >66% is blue, and >55% is gray.

A

```
                                                     Eco47III
             attB                                          M1
Δbcd       : GGGTGCCAGGGCGTGCCCTTGGGCTCCCCGGGCGCGTAGCGCT-------
M1-B2-c13  : GGGTGCCAGGGCGTGCCCTTGGGCTCCCCGGGCGCGTAGCGCTGATCGCG
B2-c13     : GGGTGCCAGGGCGTGCCCTTGGGCTCCCCGGGCGCGTAGC----------
M1-c13     : GGGTGCCAGGGCGTGCCCTTGGGCTCCCCGGGCGCGTAGCGCTGATCGCG
M1-B2      : GGGTGCCAGGGCGTGCCCTTGGGCTCCCCGGGCGCGTAGCGCTGATCGCG
c13        : GGGTGCCAGGGCGTGCCCTTGGGCTCCCCGGGCGCGTAGC----------

               HpaI  B2    EheI  c13         EcoRV attB
Δbcd       : --GTTAAC-------GGCGCC------------GATATCGGGTGCCAGGG
M1-B2-c13  : CGGTTAACGTTAAAAGGCGCCGGTAAGTAGAGTGATATCGGGTGCCAGGG
B2-c13     : -----AACGTTAAAAGGCGCCGGTAAGTAGAGTGATATCGGGTGCCAGGG
M1-c13     : CGGTT-------------GCCGGTAAGTAGAGTGATATCGGGTGCCAGGG
M1-B2      : CGGTTAACGTTAAAAGGC------------------ATCGGGTGCCAGGG
c13        : ------------------GCCGGTAAGTAGAGTGATATCGGGTGCCAGGG

Δbcd       : CGTGCCCTTGGGCTCCCCGGGCGCGTA
M1-B2-c13  : CGTGCCCTTGGGCTCCCCGGGCGCGTA
B2-c13     : CGTGCCCTTGGGCTCCCCGGGCGCGTA
M1-c13     : CGTGCCCTTGGGCTCCCCGGGCGCGTA
M1-B2      : CGTGCCCTTGGGCTCCCCGGGCGCGTA
c13        : CGTGCCCTTGGGCTCCCCGGGCGCGTA
```

B

```
             attB                                  Eco47III R3
c13        : GGGTGCCAGGGCGTGCCCTTGGGCTCCCCGGGCGCGTAGC----------
R3-c13     : GGGTGCCAGGGCGTGCCCTTGGGCTCCCCGGGCGCGTAGCGCTCGATGGA

                EheI  c13         EcoRV attB
c13        : ------GCCGGTAAGTAGAGTGATATCGGGTGCCAGGGCGTGCCCTTGGG
R3-c13     : TATGGCGCCGGTAAGTAGAGTGATATCGGGTGCCAGGGCGTGCCCTTGGG

c13        : CTCCCCGGGCGCGTA
R3-c13     : CTCCCCGGGCGCGTA
```

C

```
             attB                                  Eco47III G2
c13        : GGGTGCCAGGGCGTGCCCTTGGGCTCCCCGGGCGCGTAGC----------
G2-c13     : GGGTGCCAGGGCGTGCCCTTGGGCTCCCCGGGCGCGTAGCGCTAGCTGCT

             EheI  c13         EcoRV attB
c13        : ---GCCGGTAAGTAGAGTGATATCGGGTGCCAGGGCGTGCCCTTGGGCTC
G2-c13     : GGCGCCGGTAAGTAGAGTGATATCGGGTGCCAGGGCGTGCCCTTGGGCTC

c13        : CCCGGGCGCGTA
G2-c13     : CCCGGGCGCGTA
```

Supplementary Figure S7. Sequence alignments of constructs designed for integration into *mod(mdg4)* intron 4 (corresponding to constructs in Fig. 5C). **(A)** Multiple sequence alignment of motif combinations. **(B)** Alignment of c^{13} and *R3-*c^{13} constructs. **(C)** Alignment of c^{13} and G2-c^{13} constructs. The topmost row indicates features such as attB sites, restriction sites, and motif boundaries. Sequence similarity is color-coded: 100% identity is indicated in yellow, and >66% identity is blue.

```
                                                            C117          R1
                 |<attB                              >|      300         *
c117         : GGGTGCCAGGGCGTGCCCTTGGGCTCCCCGGGCGCGTAGCGCTGATCTGCTGTCCGATGC
only_motifs  : GGGTGCCAGGGCGTGCCCTTGGGCTCCCCGGGCGCGTAGCGCTAATCTGCTAAACGATGC
mut M & c13  : GGGTGCCAGGGCGTGCCCTTGGGCTCCCCGGGCGCGTAGCGCCAATCTGCTAAACGATGC

                     B1         R2        M                     B2      C13
                    320         *       340         *       360         *
c117         : ATATTTGTTAAATGCTGGACGGAGATCGCGCGGTTTTACACTTATTCAGTTAAAAGGTAA
only_motifs  : ATATAAGTTAAATGAAGGACGGAGATCGCGCGGTTAATTTATATAAATGTTAAAAGGTAA
mut M & c13  : ATATAAGTTAAATGAAGGACGGAGATATATATATTAATTTATATAAATGTTAAAAGCATT

                                     G2      R3
                    380         *       400         *        |<attB
c117         : GTAGAGTGATGAAATTTTAAGAAGCTGCTACGATGGATATGATATCGGGTGCCAGGGCGT
only_motifs  : GTAGAGTGTAATTATAATTATTAGCTGCTTCGATGGATATGGCATCGGGTGCCAGGGCGT
mut M & c13  : CATTCATTTAATTATAATTATTAGCTGCTTCGATGGATATTCGATCGGGTGCCAGGGCGT

                                     >|
c117         : GCCCTTGGGCTCCCCGGGCGCGTA
only_motifs  : GCCCTTGGGCTCCCCGGGCGCGTA
mut M & c13  : GCCCTTGGGCTCCCCGGGCGCGTA
```

Supplementary Figure S8. Multiple sequence alignment of constructs designed to assess the impact of spacer sequences between motifs on *mod(mdg4)* trans-splicing efficiency (corresponding to constructs in Fig. 5D). The topmost row indicates features such as attB sites, motif boundaries, and *c* subregions. Motif positions are highlighted within the alignment. Coordinates are provided relative to the intron 4 start.

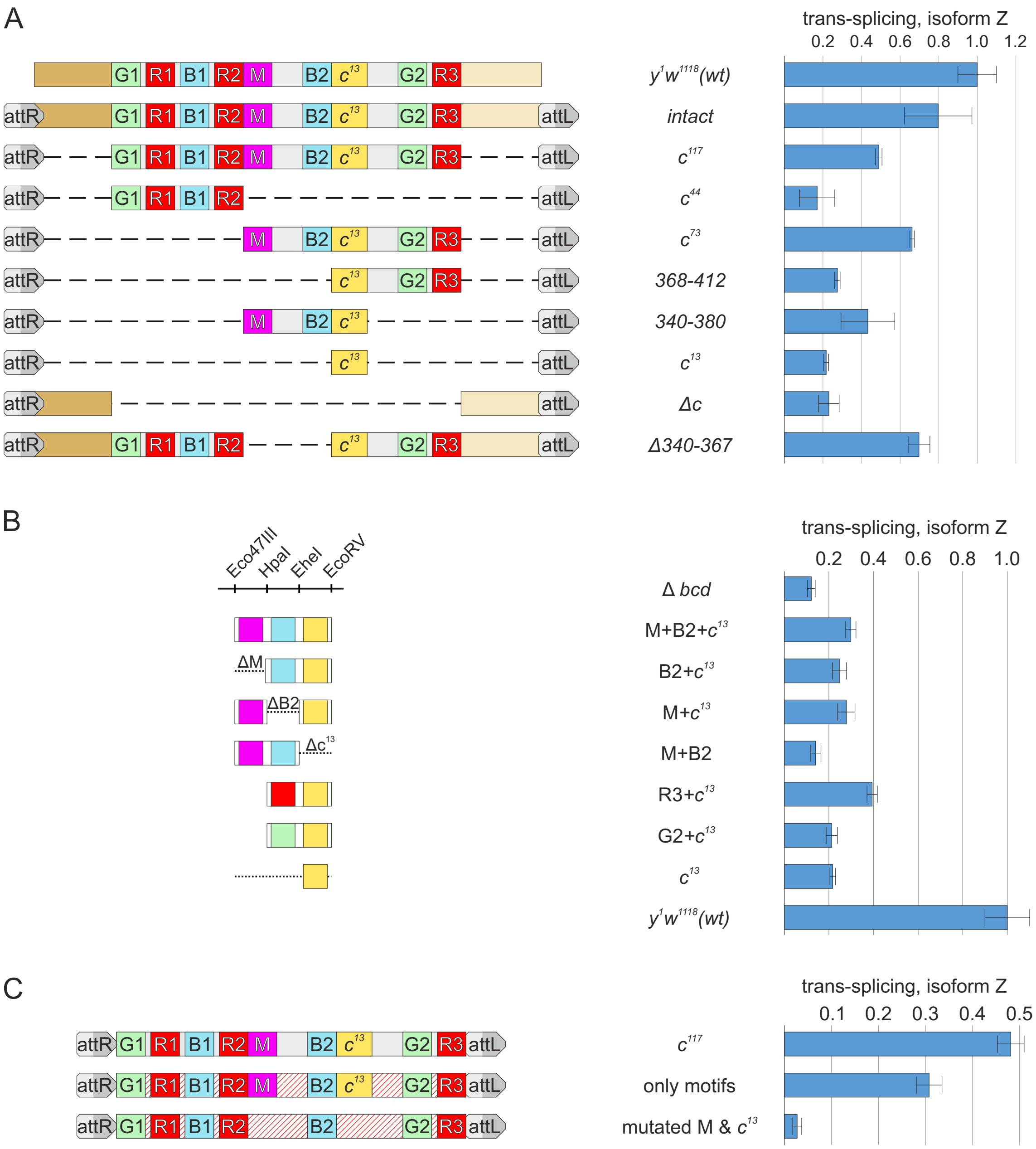

Supplementary Figure S9. Testing the role of motifs in trans-splicing between the constant region of *mod(mdg4)* pre-mRNA and the Z isoform-encoding pre-mRNA at the endogenous *mod(mdg4)* locus. **(A)** Analysis of different subregions within region *c* for their contribution to trans-splicing. The left panels illustrate tested intron fragments with assigned names; putative motifs are denoted by colored rectangles, and deletions appear as dashed lines. The right panels display trans-splicing efficiencies with pre-mRNA encoding the Z isoform. **(B)** Testing the ability of motif combinations to support trans-splicing. Trans-splicing efficiency was assessed using a 3′ exon encoding the Z isoform. **(C)** Assessment of spacer sequences between motifs on trans-splicing efficiency. Putative motifs are depicted by colored rectangles, while mutated regions are marked by diagonal hatching. Right panels present trans-splicing efficiencies measured at the pupal stage.

Each line was tested in at least three biological replicates. Error bars represent standard deviations (n = 3–18). Asterisks denote statistical significance levels: *$p < 0.05$, **$p < 0.01$, and ***$p < 0.001$.

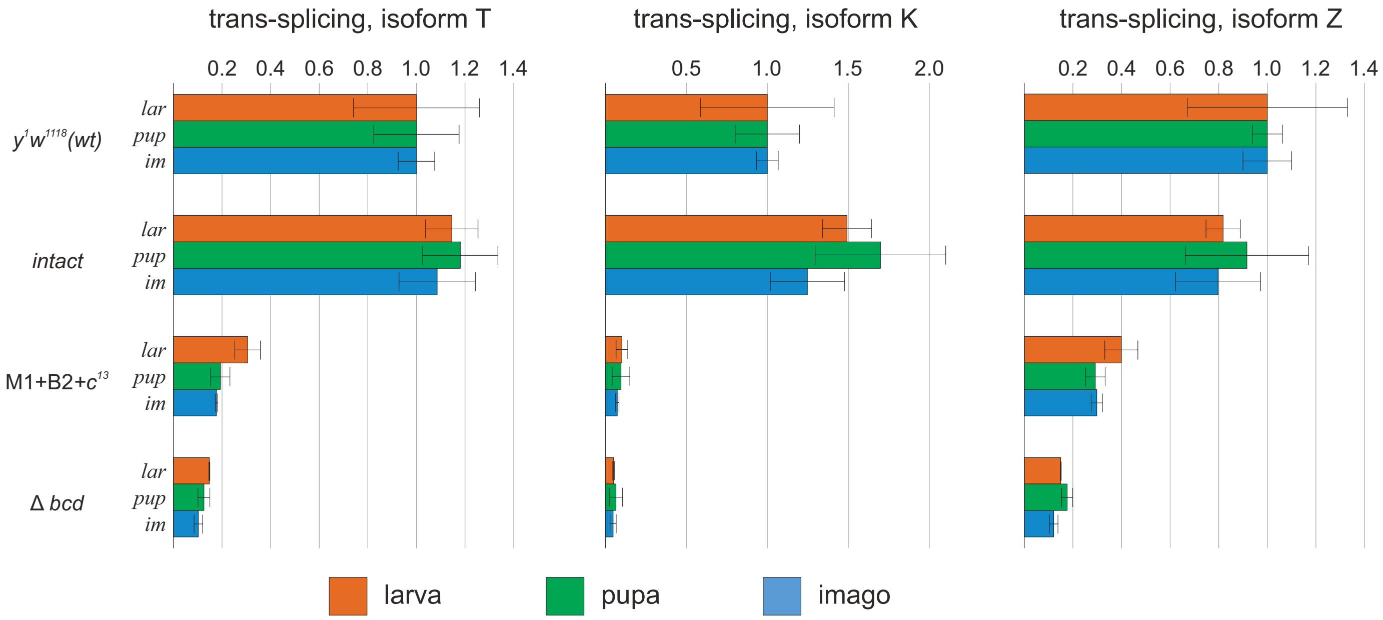

Supplementary Figure S10. Comparison of trans-splicing efficiency across larval, pupal, and adult male stages. Transgenic lines featuring *mod(mdg4)* gene mutations and the wild-type control (y^1w^{1118}) are presented on the left. On the right, trans-splicing efficiency is shown for 3′ exons encoding isoforms T, K, and Z. Each line was analyzed in at least three biological replicates. Normalization was performed using housekeeping genes (*Vha100-1* and *CG9067*), and the trans-splicing level of the control line y^1w^{1118} was set to 1. Error bars represent standard deviations (n = 3–4). Asterisks denote statistical significance: *p < 0.05, **p < 0.01, and ***p < 0.001.

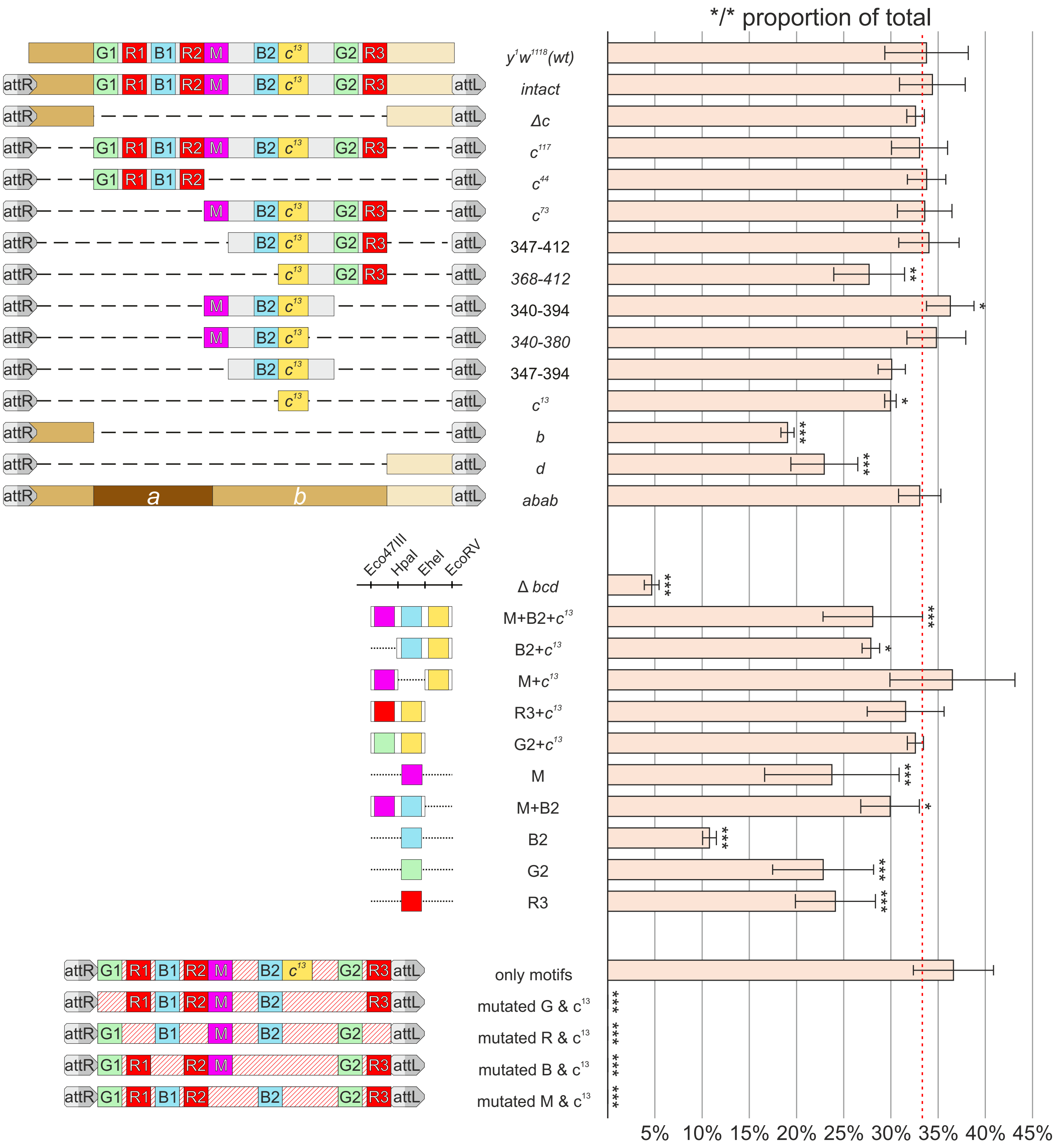

Supplementary Figure S11. Viability of the *mod(mdg4)* mutants with impaired trans-splicing. Segregation results are derived from crosses of *mod(mdg4)**/TM6 × *mod(mdg4)**/TM6, with *mod(mdg4)** denoting the modified allele. Schematics of tested intronic variants are shown on the left. Putative motifs are represented by colored rectangles, and deleted regions are indicated by dashed lines. The bar graph illustrates the proportion of homozygous *mod(mdg4)**/*mod(mdg4)** adult flies. The red-dashed line indicates the expected level of homozygote emergence from these crosses. Error bars represent standard deviations (n = 3-4). Statistical significance of phenotypic segregation ratio deviations from the expected theoretical ratio was assessed using the $\chi 2$ test: *P < 0.05, **P < 0.01, ***P < 0.001. The results of the segregation ratio analysis are provided in Supplementary Table S1.

Supplementary Table S1. Testing the viability of the *mod(mdg4)* mutants affecting trans-splicing. Segregation results derived from crosses of *mod(mdg4)**/TM6 × *mod(mdg4)**/TM6 crosses, with *mod(mdg4)** signifying a *mod(mdg4)* mutant carrying one of the construct variants. Three independent crosses were conducted for each variant. The 'number' column provides the count of flies for each phenotype, while the 'proportion' column presents their respective proportions. Mean values and standard deviations from the three crosses are also included. The expected segregation ratio was 1:2, as TM6/TM6 homozygotes are lethal. Chi-square test statistics were calculated to assess any deviation from this expected ratio. Asterisks denote significance levels: *p < 0.05, **p < 0.01, and ***p < 0.001.

mod(mdg4)*	replicate	sum	number		propotion		average value		SD		
			/	*/TM6	*/*	*/TM6	*/*	*/TM6	*/*	*/TM6	χ2 test
y1w1118	1	118	47	71	40%	60%	34%	66%	4.4%	4.4%	
	2	53	17	36	32%	68%					
	3	51	15	36	29%	71%					
	observed	222	79	143							0.477
	expected		74.0	148.0							n.s.
intact	1	318	125	193	39%	61%	34%	66%	3.5%	3.5%	
	2	314	101	213	32%	68%					
	3	224	71	153	32%	68%					
	observed	856	297	559							0.398
	expected		285.3	570.7							n.s.
Δc	1	283	90	193	32%	68%	33%	67%	0.9%	0.9%	
	2	246	79	167	32%	68%					
	3	171	58	113	34%	66%					
	observed	700	227	473							0.612
	expected		233.3	466.7							n.s.
c117	1	199	61	138	31%	69%	33%	67%	3.0%	3.0%	
	2	223	83	140	37%	63%					
	3	205	64	141	31%	69%					
	observed	627	208	419							0.932
	expected		209.0	418.0							n.s.
c44	1	275	94	181	34%	66%	34%	66%	2.0%	2.0%	
	2	258	93	165	36%	64%					
	3	225	70	155	31%	69%					
	observed	758	257	501							0.738
	expected		252.7	505.3							n.s.
c73	1	287	99	188	34%	66%	34%	66%	2.9%	2.9%	
	2	243	73	170	30%	70%					
	3	210	62	148	30%	70%					
	4	99	37	62	37%	63%					
	5	114	41	73	36%	64%					
	6	91	31	60	34%	66%					
	observed	1044	343	701							0.743
	expected		348.0	696.0							n.s.
347-412	1	197	62	135	31%	69%	34%	66%	3.2%	3.2%	
	2	218	84	134	39%	61%					
	3	212	68	144	32%	68%					
	observed	627	214	413							0.672
	expected		209.0	418.0							n.s.

mod(mdg4)*	replicate	sum	number		propotion		average value		SD		
			/	*/TM6	*/*	*/TM6	*/*	*/TM6	*/*	*/TM6	χ2 test
368-412	1	146	45	101	31%	69%	28%	72%	3.8%	3.8%	
	2	223	50	173	22%	78%					
	3	191	57	134	30%	70%					
	observed	560	152	408							1.89E-03
	expected		186.7	373.3							**
340-394	1	306	108	198	35%	65%	36%	64%	2.5%	2.5%	
	2	385	153	232	40%	60%					
	3	204	69	135	34%	66%					
	observed	895	330	565							0.025
	expected		298.3	596.7							*
340-380	1	210	80	130	38%	62%	35%	65%	3.1%	3.1%	
	2	258	79	179	31%	69%					
	3	210	75	135	36%	64%					
	observed	678	234	444							0.515
	expected		226.0	452.0							n.s.
347-394	1	299	96	203	32%	68%	30%	70%	1.4%	1.4%	
	2	307	90	217	29%	71%					
	3	215	62	153	29%	71%					
	observed	821	248	573							0.057
	expected		273.7	547.3							n.s.
c13	1	412	126	286	31%	69%	30%	70%	0.6%	0.6%	
	2	388	113	275	29%	71%					
	3	448	135	313	30%	70%					
	observed	1248	374	874							0.012
	expected		416.0	832.0							*
b	1	383	76	307	20%	80%	19%	81%	0.7%	0.7%	
	2	324	62	262	19%	81%					
	3	259	47	212	18%	82%					
	observed	966	185	781							8.72E-21
	expected		322.0	644.0							***
d	1	240	51	189	21%	79%	23%	77%	3.5%	3.5%	
	2	406	80	326	20%	80%					
	3	556	155	401	28%	72%					
	observed	1202	286	916							2.28E-12
	expected		400.7	801.3							***
abab	1	145	47	98	32%	68%	33%	67%	2.2%	2.2%	
	2	88	27	61	31%	69%					
	3	208	75	133	36%	64%					
	observed	441	149	292							0.840
	expected		147.0	294.0							n.s.
Δbcd	1	294	15	279	5%	95%	5%	95%	0.8%	0.8%	
	2	225	8	217	4%	96%					
	3	226	12	214	5%	95%					
	observed	745	35	710							9.71E-62
	expected		248.3	496.7							***

mod(mdg4)*	replicate	sum	number		propotion		average value		SD		
			/	*/TM6	*/*	*/TM6	*/*	*/TM6	*/*	*/TM6	χ2 test
M+B2+c13	1	332	78	254	23%	77%	28%	72%	5.3%	5.3%	
	2	251	89	162	35%	65%					
	3	265	67	198	25%	75%					
	observed	848	234	614							3.92E-04
	expected		282.7	565.3							***
B2+c13	1	138	40	98	29%	71%	28%	72%	0.9%	0.9%	
	2	111	31	80	28%	72%					
	3	146	39	107	27%	73%					
	observed	395	110	285							0.021
	expected		131.7	263.3							*
M+c13	1	69	30	39	43%	57%	37%	63%	6.6%	6.6%	
	2	134	37	97	28%	72%					
	3	143	55	88	38%	62%					
	observed	346	122	224							0.447
	expected		115.3	230.7							n.s.
R3+c13	1	108	37	71	34%	66%	32%	68%	4.1%	4.1%	
	2	104	36	68	35%	65%					
	3	93	24	69	26%	74%					
	observed	305	97	208							0.571
	expected		101.7	203.3							n.s.
G2+c13	1	83	27	56	33%	67%	33%	67%	0.9%	0.9%	
	2	101	34	67	34%	66%					
	3	76	24	52	32%	68%					
	observed	260	85	175							0.826
	expected		86.7	173.3							n.s.
M	1	129	21	108	16%	84%	24%	76%	7.1%	7.1%	
	2	63	21	42	33%	67%					
	3	111	24	87	22%	78%					
	observed	303	66	237							2.00E-05
	expected		101.0	202.0							***
M+B2	1	183	61	122	33%	67%	30%	70%	3.1%	3.1%	
	2	278	85	193	31%	69%					
	3	275	71	204	26%	74%					
	observed	736	217	519							0.027
	expected		245.3	490.7							*
B2	1	252	29	223	12%	88%	11%	89%	0.7%	0.7%	
	2	326	36	290	11%	89%					
	3	317	31	286	10%	90%					
	observed	895	96	799							1.11E-46
	expected		298.3	596.7							***

mod(mdg4)*	replicate	sum	number		propotion		average value		SD		
			/	*/TM6	*/*	*/TM6	*/*	*/TM6	*/*	*/TM6	χ2 test
G2	1	372	113	259	30%	70%	23%	77%	5.3%	5.3%	
	2	89	17	72	19%	81%					
	3	311	59	252	19%	81%					
	4	262	32	230	12%	88%					
	observed	1034	221	813							3.40E-16
	expected		344.7	689.3							***
R3	1	251	46	205	18%	82%	24%	76%	4.2%	4.2%	
	2	183	47	136	26%	74%					
	3	194	55	139	28%	72%					
	observed	628	148	480							2.08E-07
	expected		209.3	418.7							***
only motifs	1	106	45	61	42%	58%	37%	63%	4.2%	4.2%	
	2	231	75	156	32%	68%					
	3	186	65	121	35%	65%					
	observed	523	185	338							0.322
	expected		174.3	348.7							n.s.
mutated G&c13	1	149	0	149	0%	100%	0%	100%	0.0%	0.0%	
	2	110	0	110	0%	100%					
	3	189	0	189	0%	100%					
	observed	448	0	448							1.21E-50
	expected		149.3	298.7							***
mutated R&c13	1	155	0	155	0%	100%	0%	100%	0.0%	0.0%	
	2	142	0	142	0%	100%					
	3	204	0	204	0%	100%					
	observed	501	0	501							2.02E-56
	expected		167.0	334.0							***
mutated B&c13	1	41	0	41	0%	100%	0%	100%	0.0%	0.0%	
	2	60	0	60	0%	100%					
	3	73	0	73	0%	100%					
	observed	174	0	174							1.09E-20
	expected		58.0	116.0							***
mutated M&c13	1	176	0	176	0%	100%	0%	100%	0.0%	0.0%	
	2	80	0	80	0%	100%					
	3	152	0	152	0%	100%					
	observed	408	0	408							2.80E-46
	expected		136.0	272.0							***

Supplementary Table S2. List of primers used in this study

cloning

name	sequence	cloning method
	**transgenic model system - donor**	
prom_fw	cgaggctgtcatatgcttag	blunt
exon 4 rev	acgatgattcactgtagttctggg	blunt
IRES_fw	aggcggcgtgagagaaccaggtata	blunt
IRES_rev	cctgatcgggtatgtagaatgccac	blunt
intron 4 fw	caacagtcccagaactacag	Sal I
intron 4 rev	tgaGctccgtatctgcgaga	Sac I
grip fw	gatgctagctgtataagaccggtcgcatc	Nhe I
grip rev	gggctgcaggaattcgattcaggacgatccgcttgatcgt	Pst I
ex4 Nh_r	gcggctagccaggatcgttctgaaaaa	Nhe I
ex4 Nh_fw	gcggctagcaacgatcctgcgcctcag	Nhe I
grip ex10 Nh_r	gcggctagcgttgtatgacctgaaagg	Nhe I
grip ex10 Nh_fw	gcggctagcgtcatacaaccttgggta	Nhe I
ex3-grip i3_fw	cggatgtgagtattggtttttgg	fusion PCR
ex3-grip i3_r	aaccaatactcacatccgttagc	fusion PCR
grip ex9-in3_fw	tcgcggtgagtatatgtatacat	fusion PCR
grip ex9-in3_r	tacatatactcaccgcgacgtgc	fusion PCR
i3-grip e10_fw	tttttttttcaggtcatacaacct	fusion PCR
i3-grip e10_r	tatgacctgaaaaaaaaatattgg	fusion PCR
grip ex7 Nh fw	gatgctagctgtataagaccggtcgcatc	Nhe I
grip ex10 IRES r	gggctgcaggaattcgattcaggacgatccgcttgatcgt	fusion PCR
i2ATP_fw	gacagatcttccccatttttatgtccg	Bgl II
i2ATP_r	gacagatctAAGGGATAGCCTTTGAAACT	Bgl II
regA fw	gacagatctattgcaaagcatacatggcc	Bgl II
	**transgenic model system - acceptor**	
prom TK fw	gcgccatggagttgcagatgcggatgtgg	NcoI (Fluc)
prom TK r	gcggaattcccacgcggaatatccccg	EcoRI (mCherry)
	**genomic editing within intron-4**	
gRNA_B	CGAGAAGACCTTTCGGAAACTTAAGCTATACTTCGCGTTTTAGAGCTAGAAATAGC	BbsI
gRNA_D	CGAGAAGACCTAAACATTACTCGCTTTGCTCAGTCCGACGTTAAATTGAAAATAG	BbsI
Larm_ex4_fw	gcgtctagagaattgcccacgaaatc	Xba
Larm_i4-attP_r	gagaactcaaaggttaccccagttggggaagtatagcttaagtttgtt	fusion PCR
attP-i4_fw	cccctcgagggtgccagggcgtgcccttgggctccccgggcgcgtacgctggccattagttaataa	fusion PCR
i4 r	gtactcgagactcgctttgctcagtcaa	blunt (to EcoRV)
3xp3 fw	gcggatatcggatctaattcaattaga	EcoRV
3xp3 r	catgaattccgaccggcttcgagccga	EcoRI
mCherry_cDNA fw	GCGgaattcATGGTGAGCAAGGGCGAGGAGGATAACAT	EcoRI
mCherry_cDNA r	gcgggatccttacttgtacagctcgtccat	BamHI

SV40 poly(A)		BamHI, SalI
Rarm_attP-i4_fw	tcagtcgaccccaactggggtaacctttgagttctctcagttggggCAAAGCGAGTAATTGGAGTT	SalI
Rarm_i4_r	atagcggccgccgcgacatccgtaaata	NotI

integration within intron-4

attB-intact fw	cccctcgagggtgccagggcgtgcccttgggctccccgggcgcgtacgctggccattagttaataa	XhoI
attB-intact r	agaactagtacgcgcccggggagcccaagggcacgccctggcacccactcgctttgctcagtcaaa	SpeI
attB-Eco47 fw	CCCCTCGAGGGTGCCAGGGCGTGCCCTTGGGCTCCCCGGGCGCGTAGCGCT	XhoI
attB-EcoRV r	AGAACTAGTACGCGCCCGGGGAGCCCAAGGGCACGCCCTGGCACCCGATATC	SpeI
Green-c13 fw	CCCCGGGCGCGTAGCGCTAGCTGCTGGCGCCGGTAAGTAGAG	fusion PCR
Red-c13 fw	CCCCGGGCGCGTAGCGCTCGATGGATATGGCGCCGGTAAGTAGAG	fusion PCR
C13 r	GCCCTGGCACCCGATATCACTCTACTTACCGGCGCC	fusion PCR
340-380 fw	CCCCGGGCGCGTAGCGCTGCGCGGTTTTACACTTATTC	fusion PCR
340-380 r	GCCCTGGCACCCGATATCACTCTACTTACCTTTTAACT	fusion PCR
368-412 fw	CCCCGGGCGCGTAGCGCTGGTAAGTAGAGTGATGA	fusion PCR
c117 fw	CCCCGGGCGCGTAGCGCTGATCTGCTGTCCGATGC	fusion PCR
c117 r	GCCCTGGCACCCGATATCATATCCATCGTAGCAGCTTC	fusion PCR
M-B2-c13	ccccgggcgcgtagcgctgatcgcgcggttaacgttaaaaggcgccggtaagtagagtgatatcgggtgccagggc	fusion PCR
del bcd	ccccgggcgcgtagcgctgttaacggcgccgatatcgggtgccagggc	fusion PCR
c73 fw	gcgcggttttacacttattc	blunt (EcoRV in del bcd vector)
c73 r	atatccatcgtagcagcttc	
c44 fw	gatctgctgtccgatgca	
c44 r	gatctccgtccagcatttaa	

genotyping

transgenic model system

attB dir	GTCGACGATGTAGGTCACGG
loc22A2 rev	ACCTCGGAATGAAGGAGTGAAG

integration within intron-4

intron 4 gntp_dir	ATTCCAAACTTGAACACTTTCC
intron 4 gntp_rev	ATGCCGTTGCTGGATTT

quantitative PCR

trans-splicing

ex4 3' fw	GGATCAGGGCAACACAGAG
exT r	GCGTCTGATCCTCAACCTTT
exK r	CAGGAAGAAGTACTCATGCTTATC
exZ r	GATTGGAGCGGTACACAAAG

Fluc r	CGCCGGGCCTTTCTTTAT
mCherry r	AAGCGCATGAACTCCTTGA
splT probe	FAM-TCAAGCCGCCACATCCGCAT-BHQ1
splK probe	FAM-CCCGATGAACTGACCTTGAGC-BHQ1
splZ probe	FAM-TCAGATGTTCCTGATCAGCCGCAA-BHQ1
	**donor construct transcription**
ex4tr fw	CAACAGTCCCAGAACTACAG
grip fw	CCACGCTGACGATCAAG
IRES r	TGTTCTGGTGTCTCTCTTATTC
	**mod(mdg4) donor gene transcription**
ex4 fw	AAAGCTCCACCAGCAATC
ex4 r	AGTATCGTCCTCCACATAGG
	**normalization**
Vha100 fw	TCATCTTCCACAACGCTTAC
Vha100 r	GGAGATCCTGTTCCTGAAATAC
CG9067 fw	GGAGCAGGAACTGGAATTG
CG9067 r	TGTGGTTCTCTGTCGAGTAG
	**target enrichment**
bio-ex4	bio-ACATTTGCGGAGGTGCTTGTCTTGGA
	**template amplification for in vitro transcription**
T7_ex4	tgTAATACGACTCACTATAGGGcaacacagaggctcaa
c	atatccatcgtagcagcttc
a	attgcaaagcatacatggcc

Supplementary Table S3. The MIQE v2.0 guidelines

	PROVIDE	DESCRIPTION/JUSTIFICATION
**1. REAGENT PREPARATION**		
**Bioinformatics tools and versions and settings used to design assays**	Yes	PrimerQuest™ Tool (IDT)
**Official gene symbol, species and sequence accession number**	Yes	*mod(mdg4), vha100-1, CG9067*<br>*Drosophila melanogaster*
**Location of amplicon**	Yes	Amplicons were designed either within a single exon or spanning exon–exon splice junctions. Their locations are illustrated in the schematics; additional details can be inferred from the primer sequences provided.
**Amplicon length**	Yes	80-150 bp. Additional details can be inferred from the primer sequences provided.
**Primer and probe sequences**[c]	Yes	Supplemental Table S2.
**Location and identity of any modifications**	Yes	Modifications and their positions within the Taqman probe sequences are presented in the Supplementary Table S2.
**Manufacturer of oligonucleotides**	Yes	Lumiprobe, Moscow, Russia
**Details of optimization performed**	No	Standard protocols without optimization
**2. SAMPLE PREPARATION**		
**Detailed description of sample types and numbers**	Yes	2- to 3-day-old males generated by crossing lines with integrated Donor and Acceptor constructs<br>2- to 3-day-old males with edited intron 4 or wild-type (WT)<br>L3 larvae with edited intron 4 or wild-type (WT)<br>2- to 3-day-old pupa with edited intron 4 or wild-type (WT)<br>Generally, 15-30 flies were taken for each biological replicate.
**Sampling procedure (including time to storage)**	Yes	Samples were flash-frozen in liquid nitrogen
**Sample aliquoting, storage conditions and duration**	Yes	Samples were stored at -70℃ for no more than 7 days.
**Description of extraction method including amount of sample processed**	Yes	TRI Reagent® (MRC, Cincinnati, OH, USA) was used. Detailed information can be found in the Materials and Methods section.
**Source and amount of spike-in nucleic acids added**	Yes	Spike-in was not used.
**Volume of elution buffer used to elute/resuspend nucleic acids**	Yes	Total RNA was dissolved in 30–50 µL of DEPC-treated water

**Number of extraction replicates**	Yes	3
**Extraction blanks and percent yield included**	Yes	Not applied. Typically, 2-2.5 µg of total RNA were isolated from one fly. Percent yield was not calculated.
**Method to evaluate quality and quantity of nucleic acids**	Yes	RNA quality and quantity were assessed spectrophotometrically. The integrity of RNA from some samples was evaluated using separation by horizontal gel electrophoresis.
**Storage conditions: temperature, concentration, duration, buffer, aliquots**	Yes	Purified RNA samples, dissolved in DEPC-treated water, were stored at -70°C for no longer than 3 months.
**Clear description of dilution steps used to prepare working template solution**	Yes	The total RNA preparation was not diluted; aliquots were taken based on concentration and the requirements of the reverse transcription protocol.
**Template modification (digestion, sonication, pre-amplification, DNAse treatment etc.)**	Yes	RNA was treated with DNase I (Thermo Fisher Scientific, Waltham, MA, USA) to eliminate residual genomic DNA
**Purification after modification**	Yes	Following DNase I treatment, the sample underwent a second purification using TRI Reagent®
**3. REVERSE TRANSCRIPTION[d]**		
**cDNA priming method and primer concentration**	Yes	5 µM random hexamer
**One or two-step protocol (include reaction details for two-step)**	Yes	A two-step protocol was used. First, reverse transcription was performed to synthesize cDNA from RNA samples. Subsequently, quantitative PCR was carried out using the synthesized cDNA as a template.
**Amount of RNA used per reaction**	Yes	5 µg (RevertAid) or 1 µg (Maxima H Minus Reverse Transcriptase or LunaScript® RT Master Mix Kit (Primer-free))
**Detailed reaction components and conditions**	Yes	All reverse transcription procedures were performed according to the manufacturer's kit protocol without any modifications.
**Estimated copies measured with and without addition of RT**	No	Copy number was not directly estimated. The experiment was considered valid if no amplification was detected in the no-RT control or if amplification occurred at levels comparable to the no-template control (NTC). Additionally, the ΔCt between experimental samples and the no-RT control was required to exceed 8 cycles.
**Manufacturer of reagents, catalog number and lot number**	Yes	RevertAid Reverse Transcriptase (Thermo Scientific™) #EP0442<br>Maxima H Minus Reverse Transcriptase (Thermo Scientific™) #EP0753<br>LunaScript® RT Master Mix Kit (Primer-free) (New England Biolabs) #E3025L
**Storage of cDNA: temperature, concentration/dilution, duration, buffer, aliquots**	Yes	cDNA was diluted to a final volume of 500 µL with nuclease-free water and used immediately or stored at -20°C for up to two weeks until use.

**4. qPCR PROTOCOL**		
**Template treatment (initial heating or chemical denaturation)**	Yes	An initial step at 95°C for 2 minutes was performed for hot-start polymerase activation.
**Primer and probe concentration in the reaction and source**	Yes	Final concentration in the PCR reaction was 0.25 µM. Primers and probes were prepared as 5 µM working solutions from 100 µM stock solutions.
**Polymerase identity and concentration, $Mg^{2+}$and dNTP concentrations**	Yes	Diamant TaqA polymerase (BelBioLab, Moscow, Russia) was used at a stock concentration of 5 U/µL, with a final reaction concentration of 0.04 U/µL. The reaction mixture contained 2.5 mM $MgCl_2$ and 0.25 mM of each dNTP.
**Buffer/kit (manufacturer, catalog number and lot number)**	Yes	Diamant TaqA polymerase (BelBioLab, Moscow, Russia) with 10x Buffer (650 mM Tris -HCl (pH 8.8), 166 mM $(NH_4)_2SO_4$, 0.5% Tween 20)
**Complete thermocycling parameters including reaction volume**	Yes	Intercalating dye protocol<br>(1) 95°C, 2 min.<br>(2) 95°C, 15 sec; 60°C, 15 sec; 72°C, 30 sec.(detection stage); 40 cycles<br>(3) Melt curve<br><br>TaqMan protocol<br>(1) 95°C, 2 min.<br>(2) 95°C, 5 sec; 60°C, 30 sec; 72°C, 15 sec.(detection stage); 40 cycles
**Manufacturer and type of qPCR instrument**	Yes	QuantStudio™ 6 Flex Real-Time PCR System, 384-well
**5. DATA ANALYSIS**		
**Storage and submission of raw fluorescence data using RDES[g] or RDML[h]**	Yes	Raw fluorescence data are stored as .eds files generated by the instrument.
**Identity of standards (synthetic, plasmid, genomic, IVT[i], mRNA etc.) and method of quantification**	Yes	For housekeeping genes, a series of 1:5 serial dilutions of genomic DNA was used as standards. To determine splicing efficiency, 1:5 serial dilutions of PCR product spanning the splice junction, previously obtained by amplification from cDNA, were used as standards.
**Method of baseline correction and Cq determination**	Yes	Default software
**qPCR analysis program (source, version)**	Yes	QuantStudio™ Real-Time PCR Software v1.7.2
**Details of positive and negative controls**	Yes	As a negative control, no-RT samples were included to rule out genomic DNA contamination. For each primer pair, a no-template control (NTC) was also performed.<br><br>In experiments using the transgenic model system, the negative controls included wild-type lines lacking transgenes, as well as lines carrying only one of the transgenes—either the fully functional donor or acceptor construct. In all these cases, primer pairs designed to detect trans-splicing did not yield any amplification.

		A non-technical negative control for trans-splicing at the endogenous locus could not be established, as the absence of trans-splicing is apparently lethal.<br><br>Positive controls in the transgenic model system consisted of progeny derived from crosses between the acceptor line and the fully functional donor line. For analysis of lines with modifications at the endogenous locus, the $y^{1}w^{1118}$ (wild-type) line was used as a positive control.
**Frequency and Cq of negative controls**	Yes	For assays using TaqMan probes, no amplification was detected in no-template controls (NTCs). For assays with intercalating dye chemistry, NTCs exhibited Cq values greater than 36 cycles. Additionally, the ΔCq between experimental samples and the no-RT control was required to exceed 8 cycles.
**PCR efficiency estimation and method for its determination**	Yes	PCR efficiency was estimated using a standard curve generated from data points obtained from serial dilutions of the standards. The efficiency was calculated based on the slope of the standard curve. The measured points were within the linear range of the standard curve.
**Method of target quantity calculation**[j]	Yes	The quantity of the target in unknown samples was determined using a standard curve.
**Description of replicates**	Yes	All experimental *Drosophila* lines were analyzed using three or more biological replicates; for each line or cross, at least three independent samples were collected. Each sample was used to isolate total RNA, from which a single reverse transcription reaction was performed. The resulting cDNA was then employed for quantitative analysis. For each target gene (including both experimental targets and housekeeping genes used for normalization), four amplification reactions were run in parallel. The mean value of these four technical replicates was taken as the expression level for the corresponding biological replicate.
**Repeatability (intra-experiment variation)**	Yes	For highly expressed targets, including housekeeping genes and donor gene transcripts, the coefficient of variation (CV) for quantity ranged from 0.9% to 15%, with a mean CV of 4.9% across all samples.<br><br>For low-abundance targets, such as trans-splicing products, the CV ranged from 3.3% to 21.5%, with an average CV of 14.4%.
**Reproducibility (inter-experiment/user/lab etc. variation)**	Yes	Inter-User/Lab Variation Not Assessed
**Limit of detection calculated?**	Yes	For each primer pair, a standard dilution series was performed. The measured values were within the assay's detectable range.
**Dynamic range (limits of quantification)**	Yes	Measured values exceeded the limit of quantification.
**Method of validation of reference genes**	Yes	High-throughput sequencing data from *Drosophila* developmental stages and various tissue samples

		(flybase.org) were utilized to identify genes exhibiting the lowest standard deviation to mean expression ratio (SD/mean), reflecting the greatest stability of expression across time and tissues. An additional filtering step was applied based on expression strength, selecting genes within the same expression range as *mod(mdg4)*. The genes *vha100-1* and *CG9067* were identified and selected accordingly.
**Description of normalization method / calculation of normalized expression**	Yes	The quantity of the target gene was normalized by dividing it by the mean expression level of two reference genes, *vha100-1* and *CG9067*. Each reference gene's expression was first normalized to its average expression across all samples before calculating the combined normalization factor.
**Statistical methods used for analysis**	Yes	Student's t-test
**Choice of significance level and calculation of statistical power**	Yes	Statistical significance was determined at $p < 0.05$. In the figures, asterisks denote levels of significance as follows: *p < 0.05, **p < 0.01, ***p < 0.001.
**Specificity (when measuring rare mutations, pathogen sequences etc.)**	No	Not applicable
