# Supplementary material for: Multiple intron motifs have redundant functions in the trans-splicing of the mod(mdg4) locus
Source: Nucleic Acids Res. 2026 Aug 20;54(15):gkag823. doi: 10.1093/nar/gkag823 (PMC13490949; doi:10.1093/nar/gkag823)
Supplement: Supplementary file 1 [file gkag823_supplemental_files.zip › Supplementary Table S4 capture.docx]

**Supplementary Table S4. Mass spectrometry identification of RNA-associated proteins from S2 cell nuclear extracts.**
The table lists 188 proteins retained after filtering. Columns include gene identifiers (Gene name, FlyBase ID, and human orthologs), STRING database functional clusters, and Gene Ontology (GO) annotations (RNA-binding, spliceosomal complex, and mRNA splicing). Also provided are full protein names, normalized label-free quantification values (replicates for abc and cba RNAs), the total number of identified proteins per group, fold changes, and statistical significance (p-values).
